# Social robot design preferences as reported by stakeholders

**DOI:** 10.3389/frdem.2026.1821891

**Published:** 2026-05-18

**Authors:** Matthew Green, Dzung Dao, Sam Canning, Wendy Moyle

**Affiliations:** 1 Mechanical and Mechatronic Engineering, Griffith University, Gold Coast, QLD, Australia; 2 School of Engineering and Built Environment, Griffith University, Gold Coast, QLD, Australia; 3 School of Nursing and Midwifery, Nathan, QLD, Australia

**Keywords:** dementia, design, multi-methods, social robots, technology

## Abstract

**Introduction:**

Social robots have the potential to support older adults and their carers; but to be effective in dementia care, they must be designed in ways that align with the specific needs, preferences, and capabilities of people living with dementia.

**Objective:**

This descriptive exploratory study investigated design preferences for social robots among people with dementia and key stakeholders involved in their care.

**Methods:**

An online survey was conducted using a multi-method design involving people with dementia (*n =* 11), informal carers (*n =* 90), formal carers (*n =* 11), and healthcare providers (*n =* 4). Bayesian regression examined the hypotheses relating to preferred robot features and design attributes. Qualitative survey responses were analyzed using inductive content analysis, with exemplar quotations used to complement and contextualize quantitative findings.

**Results:**

Animal-like robots were positively received across all stakeholder groups. Telepresence robots were viewed as valuable tools for communication and information exchange, although some participants expressed concerns regarding their potential technical complexity. Both formal and informal carers reported that humanoid robots could provoke anxiety among people with dementia. Participants consistently emphasized that a robot’s capabilities and audio outputs should be congruent with its appearance, adjustable to user preferences and appropriate for the robot type. Data capture and storage of personal preferences, along with face recognition, and camera-based monitoring (infrared or skeletal tracking) were considered acceptable when they enhanced safety or supported personalized interactions.

**Conclusion:**

The findings highlight the importance of designing customizable social robots tailored to the diverse preferences and needs that shape human-robot interaction in dementia care through a co-design approach. Support for personalization, appropriate aesthetic-function alignment, and safety-enhancing data use emerged as key considerations for future social robot development.

## 1 Introduction

The global population is aging rapidly, contributing to an escalating demand for healthcare services. One of the most pressing challenges is meeting the care needs of the increasing number of adults aged 65 years and over who are living with dementia (Gale et al., 2018). This challenge is intensified by a simultaneous decline in the availability of carers (United Nations, Department of Economic and Social Affairs, Population Division, 2022), making the development and deployment of innovative technological solutions a societal priority, particularly within aged and dementia care (Kiselica et al., 2024).

Dementia is a progressive syndrome characterized by cognitive impairment that exceeds the effects of normal aging and compromises a person’s ability to live independently (Gale et al., 2018). In 2019, an estimated 52.2 million people worldwide were living with dementia, a figure projected to rise to approximately 140 million by 2050 (World Health Organization, 2021). Dementia is caused by severe neurodegenerative conditions including Alzheimer’s disease, Lewy body dementia, Parkinson’s disease, and Frontotemporal dementia (Gale et al., 2018; World Health Organization, 2017). Core symptoms include memory and communication difficulties, impaired executive functioning, and behavioral and psychological symptoms such as agitation, sleep disturbance, and anxiety (Cloak et al., 2025). Many individuals also experience co-occurring physical frailty, depression, apathy, and increased social isolation, further reducing quality of life (Koh et al., 2021).

People living with dementia in long-term care are frequently reported to have limited opportunities to engage in meaningful leisure activities or conversations that enable them to share personal histories and lived experiences (Siette et al., 2022). Insufficient cognitive, social, and emotional stimulation within these settings has been associated with poorer mood, increased loneliness, agitation, and other behavioural and psychological symptoms (Moyle et al., 2017). In response to these challenges, social robots are increasingly being introduced into long-term care settings as a means of supporting engagement and wellbeing.

Recent research has examined the potential of social robots to enhance wellbeing among people living with dementia (Wu et al., 2025; Chen et al., 2024). Social robots are electro-mechanical systems designed to support individuals with functional or cognitive impairments by facilitating social interaction, either through direct human-robot engagement or by mediating interaction between humans (Martinez-Martin et al., 2020; Ong et al., 2021; Pike et al., 2021). Existing studies suggests that social robots can provide companionship, reduce agitation and offer cognitive stimulation (Moyle et al., 2017; Chen et al., 2024). For example, social robots may offer comfort or interaction during periods when staff are unavailable, while being positioned as a complement to, rather than a replacement for, human care (Moyle et al., 2017). Furthermore, emerging evidence indicates that social robots can support engagement among people living with dementia by facilitating conversation, promoting physical and social activities, supporting reminiscence, providing comfort, and alleviating pain or distress (Moyle et al., 2019; Pu et al., 2022).

Robot type, as well as robot features and intended applications, has been shown to significantly influence both the deployment and evaluation of social robots in dementia care settings (Leng et al., 2019; Lu et al., 2021; Yu et al., 2022; Wu et al., 2025). A growing body of research has investigated how specific design characteristics shape user engagement, acceptability, and therapeutic outcomes for people living with dementia (Yu et al., 2022; Wu et al., 2025; Ghafurian et al., 2021). To date, the majority of empirical studies involving people with dementia have focused on animal-like robots, with PARO, a robotic harp seal, being the most extensively studied and widely used example (Moyle et al., 2017; Wu et al., 2025; Koh and Kang, 2018; Thodberg et al., 2016; Joranson et al., 2015). Other animal-like robots, such as JustoCat (Gustafsson et al., 2015) and RoboCat (Libin and Cohen-Mansfield, 2004), have also demonstrated benefits in engaging people living with dementia, particularly in facilitating conversation, providing comfort and entertainment, and reducing pain or distress.

In contrast, a smaller but growing body of work has examined humanoid or human-like robots in dementia care. Examples include Hiro (Sumioka et al., 2021), Telenoid (Moyle et al., 2025), Pepper (Zhou et al., 2022), and Nao (Tahan and N'Kaoua, 2023). These platforms have primarily been explored for their capacity to support verbal interaction, social engagement, and task-oriented communication. Comparative findings across robot morphologies suggest that physical form plays a crucial role in how people with dementia perceive, interpret, and interact with robotic systems.

Animal-like robots are typically designed using soft, tactile materials such as synthetic fur or silicone, incorporate touch sensors, and rely on infrared, audio, and basic movement to support communication and environmental responsiveness (Wu et al., 2025). Interaction is often facilitated through non-verbal cues, including movement, sound, and physical responsiveness (Pu et al., 2021). In contrast, some artificial-being or humanoid robots incorporate object detection, screen-based interaction, and more complex multimodal interfaces (Khosla et al., 2017), with speech recognition and verbal communication forming a primary mode of interaction (Obayashi et al., 2020; Sugiyama and Nakamura, 2022). Although the ability to communicate via natural language is widely regarded as a priority in social robot design (Hebesberger et al., 2017; Ke et al., 2020), not all effective social robots rely on spoken language. PARO, for example, produces the sound of a harp seal rather than human speech. While this sound may initially attract attention, PARO appears to exert its primary impact through movement, tactile interaction, and sustained eye contact, rather than linguistic exchange.

Accumulating evidence indicates that robot appearance plays a critical role in user acceptance and engagement. A recent review by Green et al. (2024b) highlighted the importance of robot size, shape, and materiality, noting that humanoid robots are often perceived as enabling more natural conversation, whereas animal-like robots are viewed as more “cosy” and comforting. Soft-bodied robots covered in fur or flexible materials are consistently preferred over robots with hard or mechanical exteriors. Personalization of robot design is also increasingly recognized as essential, particularly given the heterogeneous and progressive nature of dementia. In line with this, a recent review by Zhu et al. (2025) emphasized that advances in soft robotics, designed to replicate the softness and tactile sensitivity of human skin may enhance both safety and functional effectiveness when robots are used with vulnerable populations such as people with dementia.

Systematic reviews have further reported that social robots can positively influence companionship, communication, social interaction, and broader indicators of wellbeing, including mood and affect, among people living with dementia (Pu et al., 2019; Abbott et al., 2019). Nonetheless, several challenges and ethical concerns remain. These include misunderstandings or inflated expectations regarding robot capabilities, issues surrounding emotional attachment, equitable access, hygiene, and cost, as well as broader ethical considerations related to autonomy and consent (Moyle et al., 2017; Moyle et al., 2019; Ienca et al., 2016; Moyle et al., 2018; Sung et al., 2015). Addressing these concerns is essential as social robots continue to be developed and implemented in dementia care contexts.

Despite these promising developments, the full potential of social robots in dementia care remains unrealized, partly due to limited market penetration (Fracasso et al., 2022). Research on robot efficacy is also constrained by the small number of affordable devices and the lack of robots specifically designed for dementia-related needs (Ozdemir et al., 2021). Increasing the diversity of robot types could support more comparative research and advance understanding of their application within care environments.

Few studies have involved people with dementia as co-designers of social robots (Fracasso et al., 2022; Ozdemir et al., 2021) and limited research has addressed how to translate robot interventions into real-world settings (Koh et al., 2021; Wu et al., 2024). For social robots to be accepted by stakeholders, including people with dementia, their formal and informal carers, and healthcare providers, they must be perceived as useful, usable, and supportive of care needs (Chatzoglou et al., 2024). Achieving these expectations requires a holistic approach to design that considers functionality, aesthetics, personalization, and the practical realities of deployment in care settings (Chatzoglou et al., 2024).

Technology acceptance among older adults is influenced by factors such as the availability of technical support, previous technology experience, and confidence in learning new systems (Lee and Coughlin, 2015). Further, robust evidence concerning the effectiveness and appropriate duration of interventions is needed to improve understanding of social robot usability in practice (Lu et al., 2021).

A recurring limitation in social robot design research is the small sample sizes, which reduces generalizability and contributes to inconsistent findings (Koh et al., 2021). Studies comparing robot types often show a strong preference for furry, animal-like robots (Ozdemir et al., 2021; Shibata and Wada, 2011; Bradwell et al., 2021; Oh et al., 2019), although others have reported preferences for mechanically orientated humanoid designs (Pino et al., 2015). Comparative research evaluating multiple robot forms therefore provides critical insights into stakeholder preferences related to robot characteristics and appearance.

This study investigates the key design considerations required to develop practical and effective social robot prototypes for people with dementia. It extends upon a study where carers, centre managers and a person living with dementia were interviewed about their expectations and desires regarding technology for people living with dementia (Green et al., 2024a). This descriptive exploratory study explores stakeholders’ perspectives on robot type, design features, and the operational requirements for real-world deployment. A real-world feasibility framework (Bowen et al., 2009) and the principles of quality function deployment, used to translate user needs into engineering specifications (Ullman, 2016), are applied to guide interpretation and support future social robot development.

## 2 Materials and methods

### 2.1 Study aim

This exploratory descriptive study explored preferences for social robot design among people living with dementia and the key stakeholders involved in their direct formal care. Social robots were broadly defined as providing one or more of the following: social interaction, human-robot engagement, companionship, reducing agitation, and offering cognitive stimulation.

### 2.2 Study design

Data were collected from January to November 2023. A multi-method study design collected quantitative and qualitative data from multiple stakeholder groups (Beach, 2019 see supplementary materials for surveys). A review of scoping and systematic reviews informed the selection of key survey topics and shaped the theoretical rationale, which emphasized real-world feasibility, practicality, and acceptance (Koh et al., 2021; Green et al., 2024b; Daly Lynn et al., 2019). This study was guided by the Monitoring and Evaluation of Digital Health Interventions Framework (Mitchell, 2010), which advocates for the systematic assessment of feasibility, usability, efficacy, and effectiveness when evaluating complex digital health technologies. In parallel, the feasibility framework proposed by Bowen et al. (2009) was applied to ensure a comprehensive assessment across multiple domains, including acceptability, demand, implementation, practicality, adaptation, integration, potential for expansion, and limited-effectiveness testing. The combined application of these frameworks enabled a structured and theory-informed evaluation of social robot use in dementia care, with particular attention to real-world implementation considerations.

Stakeholders were invited to rate the importance of specific design characteristics related to the practical usability of social robots in care settings. These importance ratings were embedded within a Quality Function Deployment (QFD) framework, whereby stakeholder priorities inform decision matrices used to systematically evaluate and prioritize design concepts and features based on identified user needs and contextual requirements (Johnson, 2016). QFD is particularly well suited to healthcare technology evaluation, as it supports the translation of stakeholder expectations and preferences into concrete design requirements.

A second set of Likert-scale items was developed to operationalize and extend the Bowen feasibility domains by examining discrete design attributes identified in the literature as critical to the development of “useful” and contextually appropriate social robots (Lee and Coughlin, 2015; Heerink et al., 2010). These attributes included battery life, hygiene, training requirements, cost, data security and privacy, maintenance, family perceptions, upgradability, integration into routine care activities, personalization, and safety. Collectively, these items captured both technical and psychosocial factors relevant to real-world implementation, use, and sustainability.

Across all stakeholder groups, responses were recorded using a four-point Likert scale (1 = Not important, 2 = Medium importance, 3 = Important, 4 = Very important). The use of a forced-choice scale without a neutral midpoint was intended to encourage clearer prioritization of design features and reduce response ambiguity. This approach supported the identification of high-priority design and implementation factors to inform future social robot development and deployment in dementia care contexts.

Survey items were organized into four domains: (i) participant demographics, (ii) prior knowledge of and exposure to social robots, (iii) attitudes toward technologies; and (iv) design preferences and boundaries of technology acceptance.

### 2.3 Participant recruitment

The global online survey was open to English-speaking adults aged 18 and over who identified with one of four stakeholder groups: people living with dementia, informal carers (family members or friends), formal carers (registered nurses, enrolled nurses, and personal care assistants), or healthcare providers (care-center managers or national disability insurance scheme providers). These categories reflect stakeholder groups commonly included in earlier robot design studies (Koh and Kang, 2018; Moyle et al., 2025; Zhou et al., 2022; Tahan and N'Kaoua, 2023), although previous research typically includes fewer stakeholder perspectives. The survey was promoted via dementia-related organizations (e.g., Dementia Australia and StepUp for Dementia) (Pu et al., 2021), social media platforms used by carers, and public radio announcements. In addition, individuals who had previously expressed interest in participating in technology-related studies were contacted and invited to take part.

### 2.4 Ethics statement

Given the involvement of potentially vulnerable participants, all consent and information materials were written in accessible, plain language. Participants showing uncertainty or distress were to be withdrawn and where necessary, the Evaluation to Consent Measure was available to assess decision-making capacity (Resnick et al., 2007).

### 2.5 Survey instrument

The online survey included multiple-choice items, Likert scale questions, optional comment fields, and open-ended questions. Survey items were tailored to each stakeholder group’s anticipated interest and priorities. Variables were selected based on existing research and established components of social robot design (Feng et al., 2021; Hebesberger et al., 2017; Ke et al., 2020; Khosla et al., 2017). A no opinion option enabled participant to progress through the survey without pressure to provide a definitive response.

### 2.6 Statistical analyses

Bayesian analysis was employed for data analysis. Bayesian analysis which is a probabilistic framework that addresses research questions by estimating the likelihood of unknown parameters using probability distributions rather than relying on null-hypothesis significance testing (Low-Choy et al., 2017). This approach is particularly well suited to studies with relatively small sample sizes and there are unequal group sizes, as it provides more robust and informative inferences under these conditions. Unlike frequentist approaches, Bayesian analyses allow results to be interpreted using credibility intervals and probability statements, rather than *p*-values, alone, thereby, offering a more nuanced assessment of uncertainty.

Categorical variables were analyzed using Bayesian contingency tables in JASP version 0.15 (Low-Choy et al., 2017; JASP (Version 0.15), 2021). Bayesian contingency table analysis was used to compute the Bayes Factors, enabling the strength of evidence in favor of the null hypothesis of independence, as well as against it, to be quantified. Internal consistency of scale measures was assessed using Cronbach’s alpha. All additional quantitative analyses were conducted using SPSS version 29 (SPSS Statistics for Windows, Version 29.0, 2022).

### 2.7 Null hypothesis testing

The study’s null hypothesis (H_o_) proposed that all Stakeholders would hold similar opinions regarding preferred design attributes for social robots for dementia care. Stakeholder inclusion in Bayesian regression analysis was dependent on the comparability of survey questions and Likert scale structures. Bayes factors (BF₁₀) were interpreted according to Lee and Wagenmakers’ classification system (Jeffreys, 1961; Lee and Wagenmakers, 2014; Quintana and Williams, 2018), where BF_10_ 1.0 indicates no preference for either hypothesis, BF₁₀ 100 represents extreme evidence against the null hypothesis and BF₁₀ 0.01 represents extreme evidence in favor of the null hypotheses (Lee and Wagenmakers, 2014; Quintana and Williams, 2018). Sensitivity analyses were conducted with and without healthcare providers; however, due to the small number of healthcare providers (*n =* 4), comparisons were unreliable, and this group was excluded from the Bayesian analyses.

### 2.8 Qualitative analysis

Open-ended and mixed-format qualitative data were analysed using inductive content analysis as described by Maher et al. (2018). Data management, organization, and coding were supported using NVivo qualitative computer software (version 12) (Lumiverso, 2025). Analysis proceeded concurrently with data familiarization and interpretation, allowing analytic insights to inform ongoing engagement with the dataset.

Two researchers (MG, WM) independently read and re-read all qualitative responses to develop an overall sense of the data. Analytic attention was directed toward text relevant to the study aims and research questions. An initial coding framework was developed inductively from a subset of the data to capture recurrent ideas, language patterns, and meaningful concepts grounded in participants’ accounts. This preliminary coding framework was then systematically applied to the remaining dataset.

Throughout the analytic process, codes were iteratively refined, expanded, merged, or reorganized when data did not align with existing categories. Through successive cycles of constant comparison and analytic discussion, related codes were grouped into subthemes, which were subsequently organized into mutually exclusive overarching themes that reflected patterned meanings across the dataset.

Regular analytic meetings were held between the two researchers to compare interpretations, resolve discrepancies, and ensure consistency in coding decisions. These discussions supported reflexive engagement with the data and enhanced analytic rigour. Representative verbatim quotations were selected to illustrate each theme, enhancing transparency and supporting the credibility of the qualitative findings.

Qualitative findings were used to contextualise and extend the quantitative results, providing insight into participants’ perspectives without imposing predefined theoretical categories or assumptions. Member checking was not undertaken; therefore, participant feedback on the interpreted findings was not sought, and this limitation should be considered when interpreting the qualitative results.

### 2.9 Rigor and reflexivity

The first author has an established interest in the design and use of social robots for people with dementia end engaged reflexivity with this position throughout the study. He was trained and supervised in qualitative research methods by the experienced corresponding author, and all stages of survey development and implementation were conducted collaboratively under the guidance of the full author team. None of the authors had a prior relationship with participants before study commencement. Study dependability was supported using a consistent survey instrument within each stakeholder group. Confirmability was strengthened by independent initial coding conducted by two researchers (MG and WM), followed by iterative discussions to review, refine and validate the coding interpretations. Several analytic meetings were held to minimize interpretive bias, and the use of verbatim participant quotations enhanced analytic transparency. Data transferability was addressed by purposive sampling with maximum variation to capture a broad range of perspectives across stakeholder groups (Maher et al., 2018).

## 3 Results

### 3.1 Participants

A total of 216 participants commenced the online survey hosted via Lime Survey (LimeSurvey: An Open Source Survey Tool, 2026). Incomplete responses (*n =* 100) were excluded, resulting in 116 fully completed surveys retained for analysis. This level of attrition is consistent with rates commonly reported in online survey research (Eysenbach, 2004). The majority of incomplete surveys contained demographic information only; inclusion of these cases would have risked introducing systematic bias into the findings (Mack et al., 2018). Internal consistency was high, with Cronbach’s alpha indicating excellent reliability for input features (0.907) and good reliability across groups for all output features (0.895). People with dementia data was excluded due to their use of simplified scales.

Surveys with data missing completely at random (MCAR) were retained where responses were predominantly complete (typically >95%), with most of these respondents completing the full survey. This approach balanced data retention with analytic integrity.

The final sample comprised 90 informal carers, 11 people living with dementia, 11 formal carers, and 4 healthcare providers or managers (see Table 1). Across all participant groups, most respondents were female and reported advanced or higher education levels. Professional carers were predominantly aged 18 to 40 years, while family carers were most commonly aged 51 to 80 years. People living with dementia were primarily in the 61 to 80 year age bracket. Age data for healthcare providers or managers were missing.

**Table 1:** Demographics of survey participants.

Category	Informal carers (%)	Formal carers (%)	People with dementia (%)	Healthcare providers (%)
Gender				
Female	78 (86.67%)	11 (100%)	8 (72.72%)	4 (100%)
Male	12 (13.33%)	–	3 (27.27%)	–
Education				
Up to and including Year 10	2 (2.22%)	–	1 (9.09%)	–
Year 11 or 12	7 (7.77%)	2 (18.18%)	–	–
Advanced Diploma, Diploma or Cert III/IV	27 (30%)	3 (27.27%)	4 (36.36%)	–
Bachelor’s degree or higher	24 (26.67%)	4 (36.36%)	4 (36.36%)	2 (50%)
Postgraduate degree or higher	30 (33.33%)	2 (18.18%)	2 (18.18%)	1 (25%)
Missing	–	–	–	1 (25%)
Age bracket				
18–30 yrs	2 (2.22%)	3 (27.27%)	–	–
31–40 yrs	2 (2.22%)	3 (27.27%)	–	–
41–50 yrs	14 (15.56%)	2 (18.18%)	1 (9.09%)	–
51–60 yrs	24 (26.67%)	1 (9.09%)	–	–
61–70 yrs	29 (32.22%)	1 (9.09%)	3 (27.27%)	–
71–80 yrs	17 (18.89%)	1 (9.09%)	7 (63.63%)	–
81 yrs. and over	2 (2.22%)	–	–	–
Country of residence				
Ireland	1 (1.11%)	–	–	–
Israel	1 (1.11%)	–	–	–
Malta	1 (1.11%)	–	–	–
Australia	71 (78.89%)	8 (72.72%)	9 (81.81%)	4 (100%)
Russian Federation	1 (1.11%)	–	–	–
South Africa	1 (1.11%)	–	–	–
United Kingdom	2 (2.22%)	–	2 (18.18%)	–
United States	11 (12%)	2 (18.18%)	–	–
Canada	–	1 (9.09%)	–	–
Missing	1 (1.11%)	–	–	–
Income bracket (AUD)				
0–20,000	17 (18.88%)	–	5 (45.45%)	–
20,001–40,000	16 (17.77%)	5 (45.45%)	3 (27.27%)	–
40,001–60,000	17 (18.88%)	2 (18.18%)	3 (27.27%)	–
60,001–80,000	10 (11.11%)	–	–	–
80,001–100,000	7 (7.78%)	1 (9.09%)	–	–
more than 100,000	18 (20%)	3 (27.27%)	–	–
Missing	5 (5.56%)	–	–	–
Care relationship				
Caring for a partner	25 (28.89%)	–	–	–
Caring for a parent	53 (58.89%)	–	–	–
Caring for another family member	8 (8.89%)	–	–	–
Caring for a friend	1 (1.11%)	–	–	–
Other	3 (3.33%)	–	–	–
Dementia diagnosis				
Alzheimer’s disease	–	–	4 (36.36%)	–
Vascular dementia	–	–	4 (36.36%)	–
Frontotemporal dementia	–	–	1 (9.09%)	–
Vascular with Lewy body and Parkinsonism	–	–	1 (9.09%)	–
Posterior cortical atrophy	–	–	1 (9.09%)	–

Most participants resided in Australia (78.9%), followed by the United States (13.3%), with the remaining participants located in a variety of countries. Among family carers, care was most frequently provided to a parent (58.9%) or partner (27.8%). Income data were reported by family carers, professional carers, and people living with dementia, with the majority indicating an annual income in the $0 to AUD $60,000 range.

### 3.2 Design traits and feasibility criteria

Across stakeholder groups, there was strong evidence supporting the null hypothesis for several core design considerations; safety (BF₁₀ 0.006, extremely strong evidence), personalization (BF₁₀ 0.012, very strong evidence), acceptability (BF₁₀ 0.15, moderate evidence), and practicality (BF₁₀ 0.264, anecdotal to weak evidence). This evidence supports the idea that stakeholders generally agreed on the importance of those factors, rather than disagreeing.

**Figure 1:**
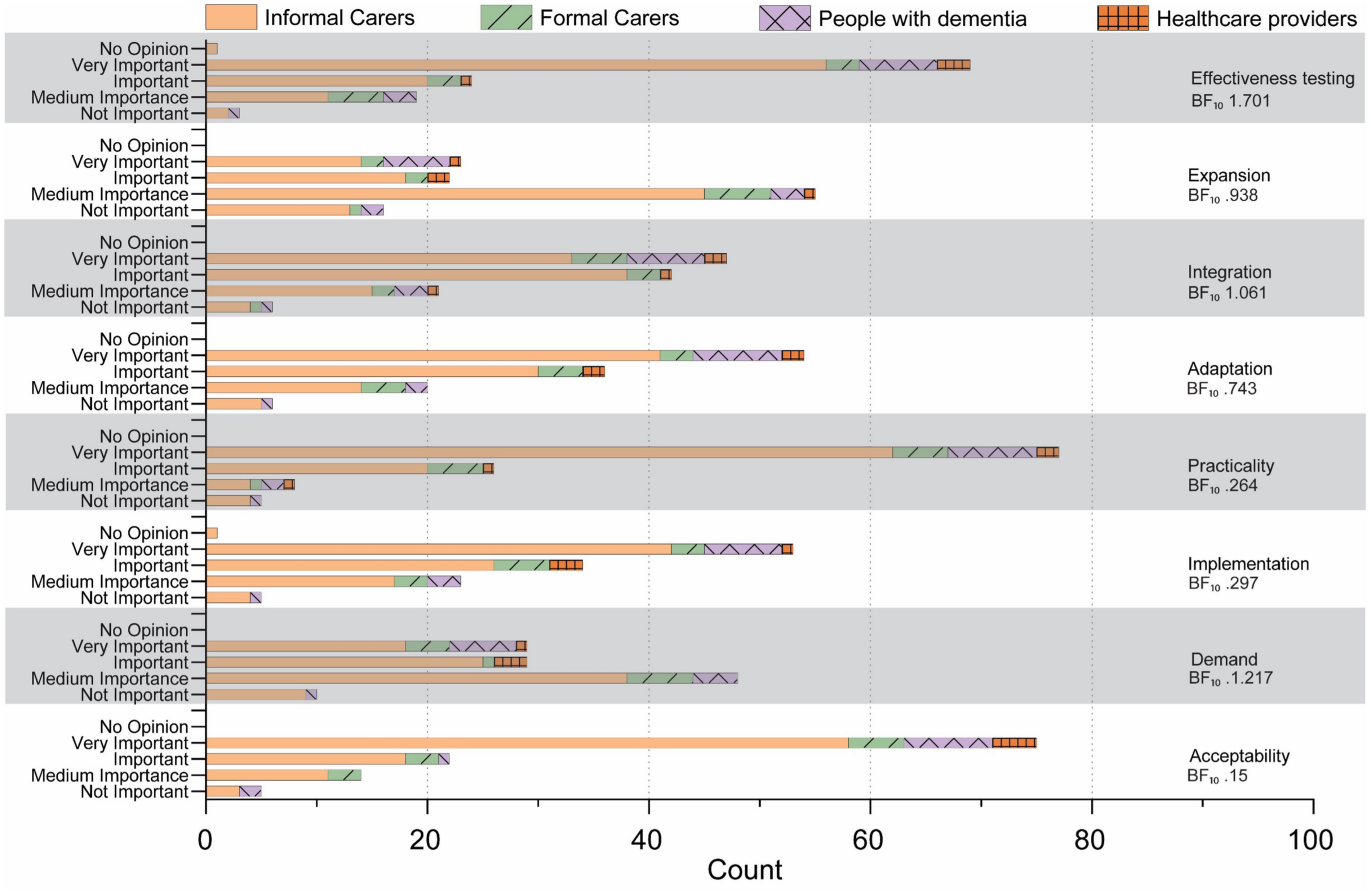
Results of Bowen feasibility criteria.

**Figure 2:**
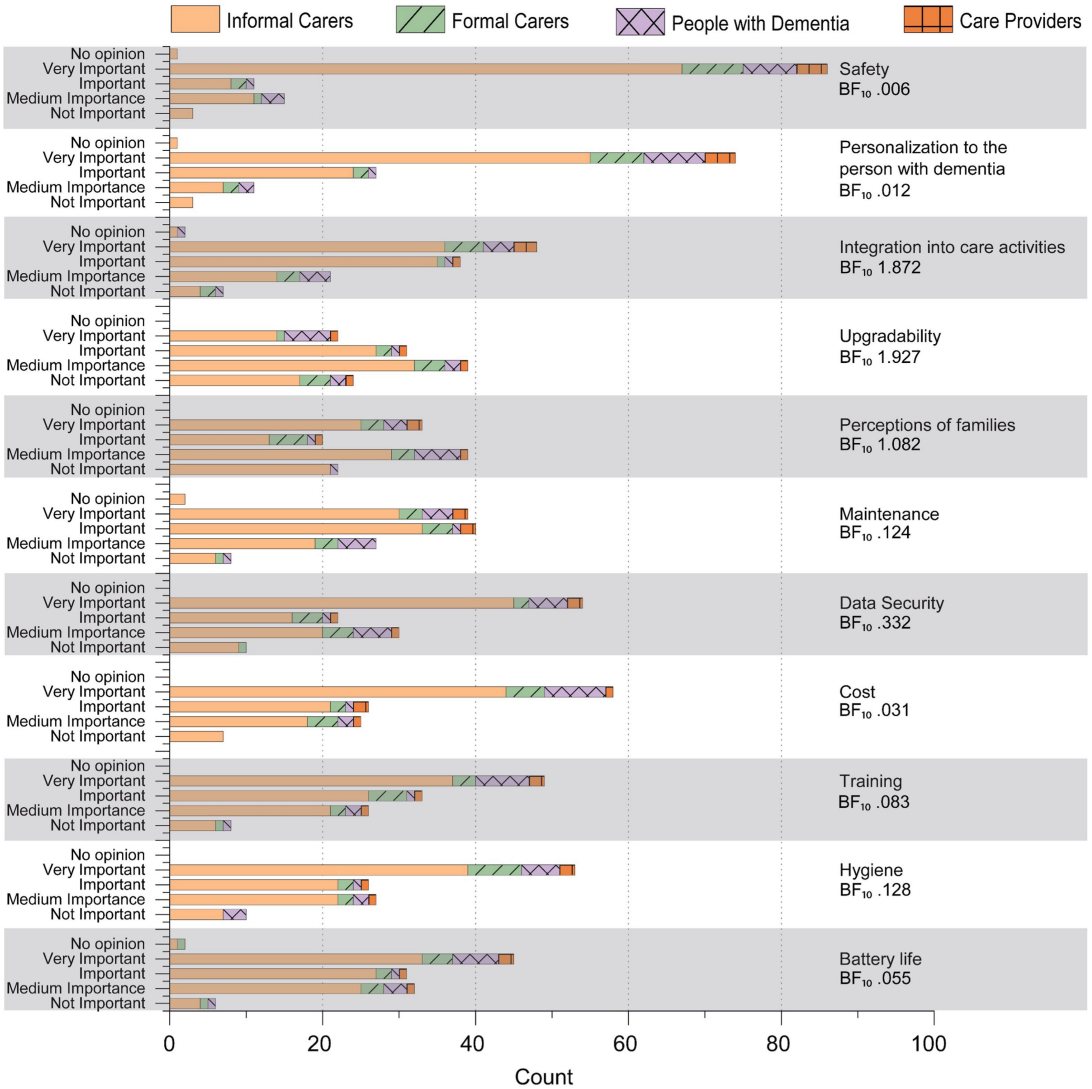
Design traits used for quality function deployment matrices.

Most design traits were rated as important or very important across all groups. However, several traits received medium to low importance ratings. They did not provide enough evidence to strongly conclude that these specific traits (demand, expansion, upgradability) are considered fundamentally different in importance across groups, including demand (BF₁₀ 1.217, evidence is anecdotal or ambiguous), expansion (BF₁₀ 0.938, evidence is anecdotal or ambiguous), and upgradability (BF₁₀ 1.927, weak evidence). Upgradability showed the greatest variability, suggesting modest disagreement among stakeholder groups. Perceptions of families produced a notably bimodal response pattern, resulting in a near-neutral Bayes factor (BF₁₀ 1.082), indicating neither strong consensus nor clear divergence (see Figures 1, 2).

### 3.3 Design features, capabilities, and appearance

Design features, capabilities, and appearance connect user-facing form with underlying engineering requirements. All stakeholder groups except healthcare, rated the importance of specific design attributes. A five-point scale (1 = Not Important to 5 = Very Important) was used for informal and formal carers; participants with dementia used a simplified three-point scale. Consequently, Bayes factor (BF₁₀) values reported below are derived from informal and formal carer data only.

Table 2 summarizes the features and capabilities rated Important to Very important for inclusion in social robot design, grouped thematically under (1) vision capture, (2) appearance features, (3) audio capture, (4) audio output, (5) touch and physiological sensing, (6) movement, with corresponding BF_10_ values.

**Table 2:** List of important features and capabilities for social robot design (BF_10_).

Important features and capabilities	BF₁₀
Vision capture	
Face position tracking without face recognition	0.406
Face recognition to detect emotional states (e.g., happy or sad)	0.166
Face recognition to detect pain	0.128
Light-sensing technology for complex sleep-tracking functions	0.1
Formal carers placed slightly lower importance on facial-recognition capabilities	1.811
Appearance features	
Moving eyebrows	0.093
Smiling or frowning mouth	0.141
Blinking eyes	0.281
Audio capture	
Pitch/tone analysis to interpret user emotion (e.g., happiness or anger)	0.132
Speech recognition for verbal communication	0.95
Song recognition for sing-along interactions	0.09
Song recognition for personalized playback (e.g., downloading and playing preferred music)	0.12
Audio output	
Barking sounds for dog-like devices	0.807
Verbal communication (humanoid/mechanized robots)	0.14
Music outputs for robots resembling living entities	0.334
Music output for mechanized (non-humanoid) robots	0.22
Touch and physiological sensing	
Stroking/animal-patting sensors	0.64
Temperature sensor (information relayed to the carer)	0.105
Temperature sensor (internal use to detect discomfort)	0.053
Heart-rate monitor (information relayed to carer)	0.376
Heart-rate monitor (internal use to detect agitation/pain)	0.243
Movement	
Acceleration sensing for measuring aggression	0.096

### 3.4 Predicted interaction time

Stakeholders estimated likely interaction durations with a social robot, a parameter that informs battery capacity (and thus device weight) and the variety/frequency of interactions needed to sustain engagement (Heerink et al., 2010). Response options were 0–5, 6–10, 11–15, 16–20, and >25 min.

More than one-quarter of informal carers (28%, *n =* 90) and 36% of formal carers (*n =* 11) anticipated that people with early-stage dementia would engage for > 25 min. Participants with dementia were more conservative; 27% indicated a maximum interaction time of 21–25 min.

### 3.5 Exposure to social robots

Prior exposure to technology is a known determinant of perceptions and acceptance (Wang et al., 2017). Three of four healthcare providers reported that social robots had been used by staff under their supervision. Prior experience was substantially higher among formal carers (54.55%) than informal carers (8.88%), though the small formal carer sample (*n =* 11) warrants caution in interpretation. Among informal carers with social robot experience (8.88%), only 1.1% had received social robot-specific training.

### 3.6 Training preferences

Informal carers, formal carers and healthcare providers rated their preferred training modalities of social robots (1 = least preferred to 5 = most preferred) online videos, online interactive (virtual) training, and in-person training. Across all groups, in-person training was most preferred, online interactive training was moderately preferred, and online videos were least preferred.

### 3.7 Qualitative findings

Three qualitative themes were generated, (1) robot acceptance, (2) data capture and storage, (3) individual or shared devices.

#### 3.7.1 Theme 1: robot acceptance

**Figure 3:**
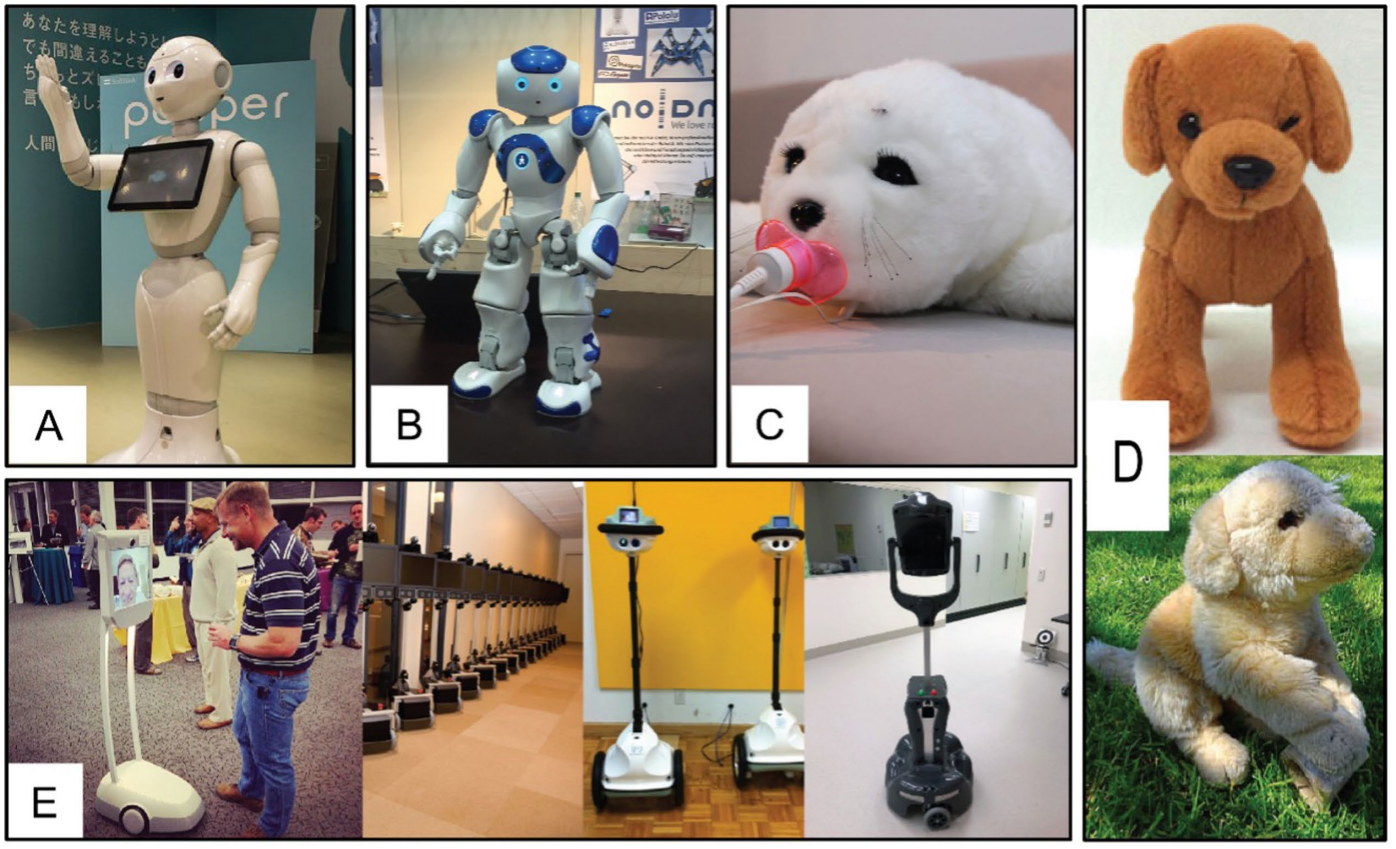
Images used for views on robot acceptance. **(A)** Pepper **(B)** NAO **(C)** PARO **(D)** Animal-like **(E)** Telepresence.

**Figure 4:**
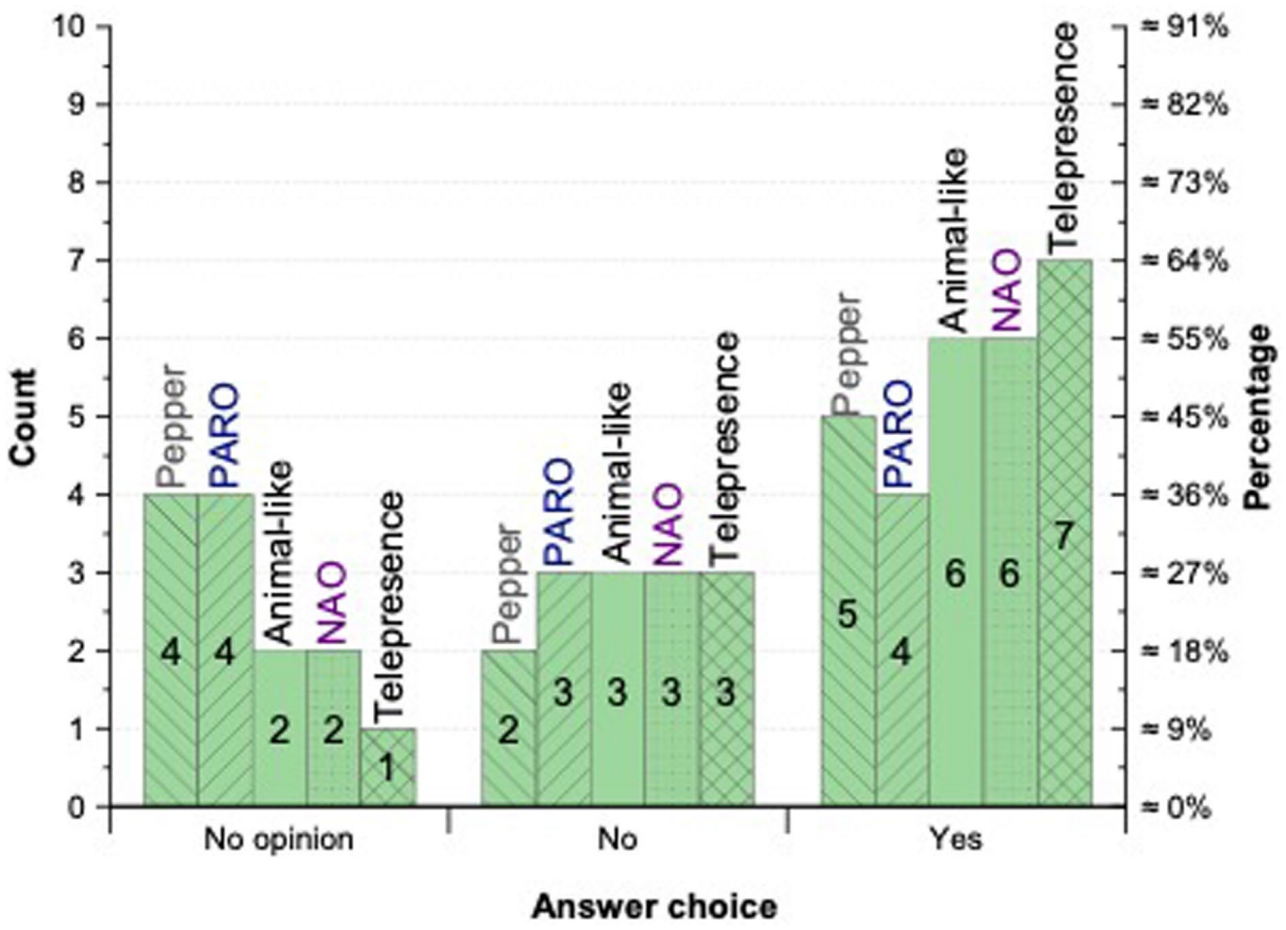
Acceptance of robot types by people with dementia.

**Figure 5:**
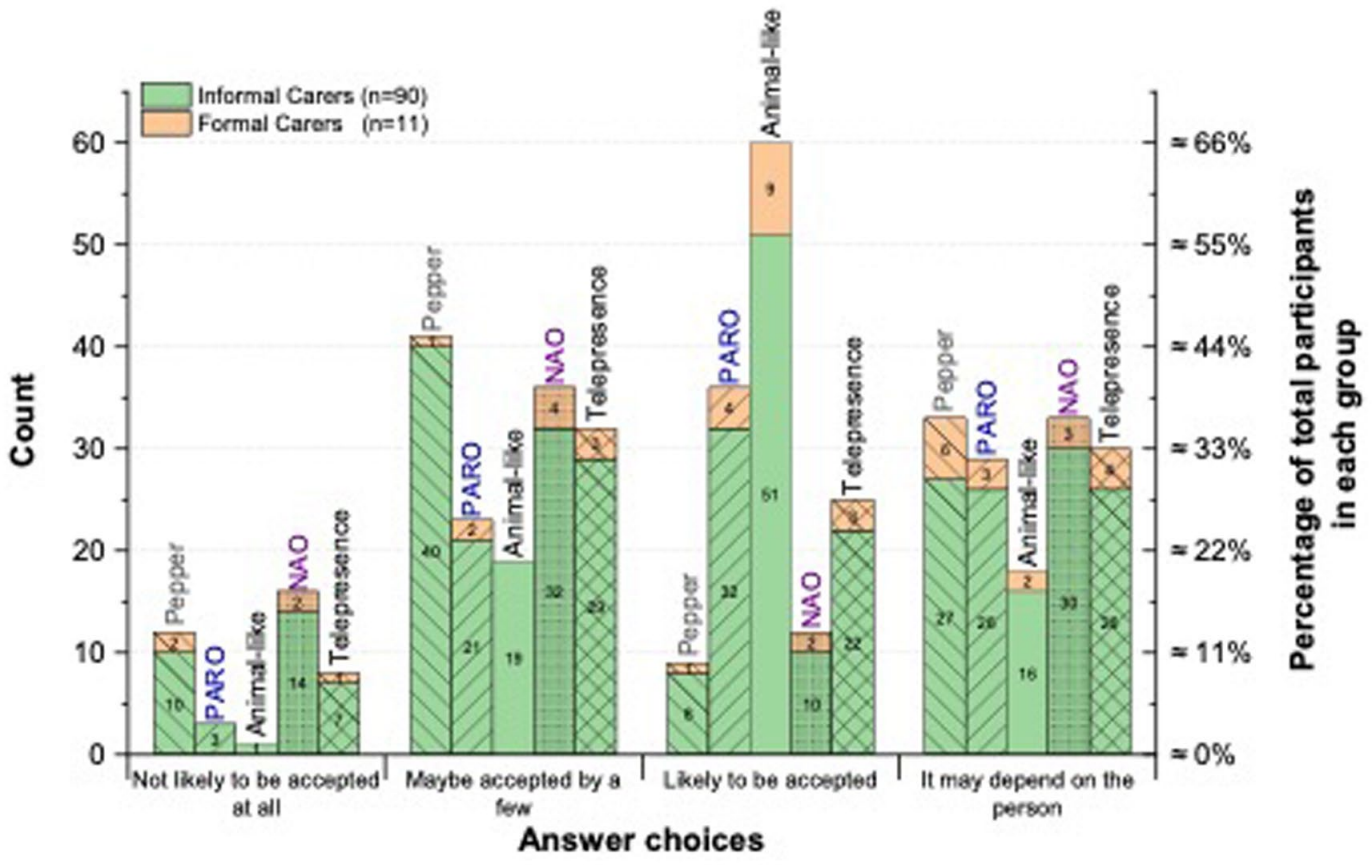
Informal and formal carers’ opinions of robot acceptance by people with dementia.

To examine which robot types are most likely to be accepted by people living with dementia, participants were asked to predict the acceptance of five robot types: Pepper, NAO, PARO, animal-like, and telepresence robots (Figure 3). Each robot type was introduced with a brief description before participants were asked: “*Based on your experience, would the following social robot technology be accepted by people with dementia*?” For example, Pepper was described as: *“Pepper is a humanoid robot capable of autonomous navigation, limb movement, and speech recognition with verbal dialogue. Pepper also has touch sensors, a display screen for visual information, and cameras that can recognize, then interact with the user.”* Formal and informal carers responded using a four-point Likert scale: 1 = Not likely to be accepted at all; 2 = Maybe accepted by a few; 3 = Likely to be accepted; 4 = It may depend on the person (see Figures 4, 5).

An open-ended comment box followed each item to allow elaboration. Participants living with dementia were asked a simplified version of the questions. For each robot type, they indicated whether they would personally accept the robot by selecting Yes, No, or No opinion.

Informal and formal carers generally judged animal-like robots to be the most acceptable option for people with dementia, relative to other robot types. Among participants with dementia, six of 11 indicated they would personally accept an animal-like robot. One participant (#15) remarked, *“Very much positive feelings and memory recall to have this for people who always had pet dogs/or even liked having a pet dog.”* Informal carers expressed similarly favorable views, while noting that animal-like forms could provoke anxiety in individuals uncomfortable with dogs. By contrast, formal carers were consistently optimistic about the potential of animal-like robots.

Only four participants with dementia reported that PARO would be “likely to be accepted.” Two participants described PARO as *“too infantile,”* (#15) and *“Not age-appropriate”* (#24). These concerns contrasted with a carer’s endorsement (#32), characterizing PARO as *“cute, cuddly, and non-threatening.”* Several carers also observed that PARO and similar devices are sometimes perceived as toys rather than as social robots intended for therapeutic psychosocial support.

Telepresence robots received the highest acceptance among participants with dementia. People with dementia and carers alike emphasized their value for communication and information exchange. As one participant with dementia (#15) explained:

*We need to have them in residential aged care for all, and more than one. They need to be well balanced to the ratio of the residents so the person/resident can directly interact with their GP. Also, there needs to be a third-party family member/nominated support person in the teleconference.*

Informal and formal carers frequently voiced concerns that humanoid robots such as Pepper and NAO might frighten people with dementia or contribute to anxiety. Many perceived these platforms as technically complex and potentially frustrating to operate. Overall interest was tempered by caution. Reflecting this nuance, a participant with dementia (#16) offered a more positive view of Pepper: *“I am a dementia sufferer and have no experience with this equipment, but it would be better than a pretend seal (PARO).”*

Conversely, an informal carer (#32) commented: *“My wife no longer has any comprehension of any form of technology. She would probably be frightened of this” and* suggested that a familiar animal-like robot would be better received. Another participant with dementia (#19) noted: *“This (NAO) may not be accepted by as many people living with dementia as the cute and cuddly type technology but would be well received by some.”* Similarly, a professional carer (#21) observed: *“I think NAO might intimidate some people with dementia (especially those in later stages) but might be regarded with curiosity by those in the early to middle stages of dementia.”*

#### 3.7.2 Theme 2: data capture and storage

Data capture and storage are central to the design and deployment of social robots. Stakeholders evaluated acceptability of data practices using the options “not acceptable,” “borderline acceptable,” “acceptable,” or “no opinion.” Participants with dementia used a simplified scale without the “borderline acceptable” option.

There was broad consensus that skeletal and infrared tracking are acceptable. People with dementia and informal carers underscored the importance of consent, and many prioritized safety-related data retention over privacy concerns. All groups deemed data retention for personalization acceptable. Healthcare providers generally viewed data retention favourably, with one exception: monitoring a person with dementia “*regardless of the risk of self-injury*,” which they rated borderline acceptable.

Qualitative comments revealed thematic differences between stakeholder groups. Healthcare providers and formal carers emphasised privacy and consent rights, whereas informal carers and participants with dementia endorsed data collection for safety and personalisation. Illustrative comments from an informal carer (#5) stated: *“Monitoring of paid care in residential aged care, that is, regular spot checks to show that people are being treated with respect and dignity.”* Another informal carer (#78) supported data retention that would be “*saved and available to medical staff to assist in planning*. A participant with dementia (#9) added: *“It (data) should be able to detect elder abuse. Have a visual and audio recording for just-in-case situations, staff training, and monitoring.”*

#### 3.7.3 Theme 3: individual or shared devices

Formal carers and healthcare providers were asked whether social robots should be individually assigned or shared. One healthcare provider (#12) noted, “*Depends on the device and the person’s attachment to it,”* and a formal carer (#21) similarly commented, *“It depends on the person (with dementia).”* Aside from these conditional responses, stakeholders strongly favored individual assignment citing benefits including a sense of ownership, reduced conflict among residents, and better end-user fit. Formal carers also highlighted practical advantages: easier personalization without repeated reprogramming, lower cognitive load for the carer, fewer interpersonal disputes and improved infection control.

## 4 Discussion

Social robots are likely to increase in use in the future; therefore, it is imperative that they have an appropriate design for the population they are intended for. This research provides exploratory, descriptive, practical and comprehensive design insights across a broad range of topics and stakeholder groups. Participants’ perceptions are important in robot design and co-design of social robots will ensure they are accepted and meet user needs. Designing robots that meet the needs of older adults living with dementia offers the opportunity to enable robots to establish long-term, non-threatening, and engaging communications, reduce agitation, and provide comfort. Social robots design that make them appear friendly and maintain social norms will enhance opportunities that they provide to older adults. Understanding design issues ensures that robots respect personal space and manage user data to mitigate privacy and foster trust. Participants, however, expressed concern about the technological complexity of telepresence robots and humanoid robots were the least preferred robot. Appropriate training and ongoing support may help alleviate these concerns.

The application of Bayesian analysis in this study aimed for meaningful comparison of quantitative data across the stakeholder groups, however, the imbalance in the sample numbers did not allow a comparison of all stakeholder groups. Qualitative responses complemented and enriched the quantitative findings, providing deeper contextual understanding of the design requirements for social robots intended to support people living with dementia. No evidence emerged of divergence between combined informal and formal carers, who responded similarly and could be treated as a combined stakeholder group.

The design features reported as being most important by stakeholder groups were safety of robots, personalization and acceptability. The literature also supports these design features as being important in the deployment of social robots into aged care (Yu et al., 2022; Wu et al., 2025; Ghafurian et al., 2021). Safety can be divided into physical and perceived safety with the key features of safety reported as comfort, experience or familiarity, predictability, sense of control, transparency and trust (Akalin et al., 2022). However, safety perception of users has often been overlooked and can result in challenges in relation to robot acceptance (Salvini et al., 2021).

Participants showed interest in personalized social robots. Although ambiguity surrounds the word “personalization,” all stakeholder groups emphasized its importance. This aligns with previous research highlighting personalization as a critical factor in social robot acceptance among people with dementia (Heerink et al., 2010; Wang et al., 2017; Bradwell et al., 2019; Robinson et al., 2013). Some studies suggest that personalized aesthetics enhance acceptance of social robots (Ke et al., 2020), while others emphasize tailoring interactions and responses (Whelan et al., 2018). One way to operationalize personalization may be to design social robot platforms capable of adapting their functionalities over time as the user’s needs evolve or programming them to provide individualized support.

Recent advances in artificial intelligence (AI) have advanced personalization of social robots (Rincon Arango et al., 2025). Advances in AI can help make social robots more user-friendly for vulnerable groups such as people living with dementia. Such robots can reduce loneliness, provide cognitive support and manage the individual tasks of older people living with dementia (Rincon Arango et al., 2025). Enabling facial recognition, recalling previous conversations, and speaking multiple languages can offer a personalized connection.

Survey responses and open-ended comments indicated that humanoid robots, like Pepper and NAO, were perceived as difficult to use and likely to require specialized training. Consequently, participants reported that they were less inclined to support the use of humanoid robots in dementia care. In contrast, animal-like robots were considered easier to use and less likely to induce anxiety in people with dementia. Their aesthetic quality was consistently regarded as more acceptable to stakeholders, positioning animal-like robots as a promising design direction.

Although the findings support animal-like robots as a design that participants preferred, the findings also underscore that no single type of animal-like robot will be universally accepted by people with dementia (Moyle et al., 2017; Wu et al., 2025). Designers must be mindful that some individuals have aversions to animals, making certain forms unsuitable. While the animal-like robot PARO, for example, does not appeal to everyone, it has several advantages: people with dementia have not usually had a bad experience with harp seals, its size and shape make it easy to hold, its synthetic fur is soft and easy to clean, it is suitable for shared use, and it interacts through vocalizations, nudging and gaze behaviors. The principal limitation of PARO is its cost. At AUD $8,500 it remains prohibitively expensive for many organizations and individuals. Cost considerations are critical, as aged care providers and older people often have limited budgets for advanced technologies (Rincon Arango et al., 2025). Future robot studies must include cost effectiveness evaluation so that potential users can understand their cost and use this information in their purchase decisions (Mervin et al., 2018).

Perceptions of importance increased when participants were provided with a clear purpose for design features, capabilities, or visual attributes. These findings support the existing literature related to the Almere model of technology acceptance, in which “perceived usefulness” is a pivotal determinant of technology adoption (Heerink et al., 2010; Whelan et al., 2018). Increasing uptake of social robots may therefore depend on effectively communicating their benefits and capabilities to stakeholders.

It is commonly assumed that the acceptability of social robots in care settings will naturally increase over time as familiarity with the technology grows. However, the findings of this study highlight a disconnect between this expectation and current practical realities. Despite optimism about future acceptance, participants identified their expectations of the use of social robots, and such expectations may contrast with limited battery life of existing social robots. Although users were not expected to engage with social robots for prolonged continuous periods, the ongoing requirement for recharging, especially if the robot was used by multiple users could undermine reliability, availability, and perceived usefulness (Deshmukh et al., 2018). This practical limitation challenges assumptions of seamless integration and suggests that technological shortcomings may temper anticipated gains in acceptance. Unless these constraints are addressed, user expectations of social robots may remain misaligned with actual everyday performance, potentially limiting sustained adoption and meaningful use in care settings.

Survey responses also revealed surprisingly permissive attitudes toward surveillance and data retention among several stakeholder groups. This finding is noteworthy given that social robots inherently raise privacy risks due to continuous data capture via cameras, microphones and other sensors. Such data collection presents ethical concerns in dementia care, where obtaining ongoing, informed consent may be challenging due to cognitive impairment and fluctuating capacity. In many contexts, privacy concerns have been identified as a key barrier to the adoption of social robots. However, in the present study, participants reported greater acceptance of data collection when it was clearly linked to a defined, beneficial purpose, such as enhancing safety, care quality, or engagement.

Stakeholders appeared willing to tolerate data retention when the perceived advantages outweighed potential privacy infringements, suggesting a pragmatic trade-off between risk and benefit. In contrast, healthcare providers expressed more cautious attitudes toward surveillance and data storage, highlighting heightened awareness of professional, ethical, and legal responsibilities associated with safeguarding sensitive information. These concerns warrant particular attention, as healthcare providers are often key decision-makers in technology adoption and implementation. Their perspectives underscore the importance of incorporating robust privacy protections, transparent data-governance strategies, and clear accountability mechanisms into the design of data-capturing robotic systems.

Collectively, these findings emphasize the centrality of trust in the deployment of social robots and the need to ensure that sensitive data are protected against unauthorized access or misuse. They also point to the importance of contextual factors in shaping privacy perceptions. In line with the findings of Jayaraman et al. (2024), who demonstrated that privacy concerns are more strongly influenced by the task performed and the context in which a robot operates than by robot appearance alone, our results suggest that ethical acceptability may depend less on robot design and more on how, why, and by whom data are collected and used. This highlights the need for further empirical investigation into task and context-specific privacy expectations, particularly in dementia care settings, to inform ethically grounded design and implementation strategies.

Furthermore, limited exposure of carers to social robots and in particular, limited access to structured training may significantly constrain market adoption, especially if broader acceptance in home-based care is a strategic objective. Across stakeholder groups, in-person training was identified as the preferred mode of learning, reflecting the perceived need for hands-on experience and guided interaction to build confidence and competence in using social robots. However, reliance on in-person training presents substantial practical challenges. Such training models are costly to fund, resource-intensive to deliver, and difficult to scale, particularly when multiple sessions are required to support skill acquisition, confidence, and ongoing use. In addition, logistical barriers, including time constraints, workforce availability, and geographical dispersion may limit attendance and sustained participation. These tensions highlight a critical disconnect between the recognised importance of training for successful implementation and the feasibility of delivering in-person training at scale, underscoring the need for more flexible, accessible, and scalable training approaches to support long-term adoption.

Finally, the survey findings support the need for co-design. When co-design involves all stakeholders, the resulting product can benefit their needs and preferences. While co-design has been embraced it is not effective unless older people, people with dementia and their carers are involved (Moyle et al., 2025).

### 4.1 Strengths and limitations

A key strength of this study is its global survey design, informed by evidence-based literature and underpinned by a theoretical rationale emphasizing real-world feasibility, practicality, and user acceptance. This approach enabled the inclusion of diverse stakeholder perspectives and ensured that the findings meaningfully reflect design needs and preferences relevant to the development of social robots for people living with dementia. The use of content analysis further strengthened the study by grounding interpretations directly in participants’ data and perspectives.

Several limitations should be acknowledged. Recruitment of this population is inherently challenging, particularly when participation involves survey completion. To maximise participation, recruitment was conducted widely using social media and established dementia research platforms. While this approach facilitated efficient recruitment, it may have introduced selection bias, as individuals who engage with online research recruitment platforms or respond to social media advertisements may be more interested in, familiar with, or receptive to technology than the wider population. Consequently, the sample may not fully represent individuals with lower levels of digital literacy or interest in technological interventions. This potential bias should be considered when interpreting the findings, particularly with respect to the generalisability of results to broader community-based populations. Future studies may benefit from complementary recruitment strategies, including direct community-based sampling, to enhance representativeness.

To maximize accessibility, simplified response options were provided for participants living with dementia. While appropriate given cognitive considerations, this limited the comparability of their responses across stakeholder groups. The use of consistent but flexibly adapted response scales may have supported more robust comparative analyses without compromising inclusivity.

Survey feasibility requirements also necessitated the presentation of a limited set of exemplar social robots within each category. Although this aimed to reduce participant burden, it may have constrained the breadth of robot types considered and, consequently, the diversity of design preferences captured.

The sample was unbalanced, particularly with respect to the small number of participants living with dementia, which limited the strength of group comparisons. Recruitment focused on community-dwelling individuals to align with the study’s emphasis on independent living and cognitive contribution; however, broader recruitment, including residential aged care or nursing home settings, may have enhanced participation and representativeness.

The study was further affected by substantial missing data, largely attributable to survey abandonment after completion of demographic items, likely reflecting perceived survey length. Future research should consider shorter or staged survey designs to improve completion rates.

Finally, the study intentionally prioritized the consumers and care-focused perspectives, excluding participants from information technology or robotics disciplines. While this strengthened the relevance of findings to lived experience and care priorities, it limited the inclusion of technical viewpoints. Additionally, despite efforts to recruit broadly, medical and allied health practitioners were not represented, and some clinical perspectives may therefore be underrepresented. The reliance on written qualitative responses also limited opportunities for peer debriefing, observation and member checking (Manning, 1997).

Future research would benefit from larger and more diverse samples of people living with dementia, inclusion of interdisciplinary professional technical expertise, and mixed methods to further inform the design and implementation of social robots.

## 5 Conclusion

This survey offers preliminary evidence on stakeholder-informed design preferences for social robots intended to support people living with dementia. Despite acknowledged limitations, the findings demonstrate a consistent preference for animal-like robots across all participant groups, underscoring their perceived acceptability and emotional resonance. Telepresence robots were valued for their potential to facilitate communication and social connection, although concerns were raised regarding their technological complexity. Humanoid robots were the least preferred, suggesting misalignment between their form and the needs or expectations of users in dementia care contexts. Across stakeholder groups, personalization emerged as a critical design priority, highlighting the need for adaptable and user-centered approaches to robot design.

Collectively, these findings emphasize that social robot design for dementia care should prioritize familiarity, simplicity, and individual relevance over technological sophistication alone. Future research should extend this work through participatory and co-design methodologies that meaningfully involve people living with dementia alongside carers and professionals. Such approaches are essential to translating user preferences into ethically grounded, feasible, and acceptable social robotic solutions capable of supporting independence, wellbeing and quality of life.

## Data Availability

The raw data supporting the conclusions of this article will be made available by the authors, without undue reservation.
