# Supplementary material for: Social robot design preferences as reported by stakeholders
Source: Front Dement. 2026 May 18;5:1821891. doi: 10.3389/frdem.2026.1821891 (PMC13222834; doi:10.3389/frdem.2026.1821891)
Supplement: Supplementary file 3 [file Supplementary_file_3.pdf]

Family carers input towards the design of social assistive technology

This research aims to design social assistive technologies for people with dementia. An important aspect of this research is how to help carers do their job by designing for real-world scenarios. Consequently, the opinions of carers are highly valued.

Family Carers input for "Bespoke social-assistive technologies for people with dementia: A user-driven approach."

Basic Demographics

[]Gender

Choose one of the following answers

Please choose **only one** of the following:

- ◯ Female
- ◯ Male

- ◯ Transgender
- ◯ Gender not specified

[]
What is the highest level of education you have achieved?

Choose one of the following answers

Please choose **only one** of the following:

- ◯ Up to and including Year 10
- ◯ Year 11 or 12
- ◯ Advanced Diploma, Diploma or Cert III/IV
- ◯ Bachelor’s degree or higher
- ◯ Postgraduate degree or higher

[]
Check the age bracket you belong in?

Please choose the appropriate response for each item:

	18-30yrs	31-40yrs	41-50yrs	51-60yrs	61-70yrs	71-80yrs	81yrs and over
Age	◯	◯	◯	◯	◯	◯	◯

[] Which of the following best describes your care relationship with the person you care or previously cared for?

Choose one of the following answers

Please choose **only one** of the following:

- ◯ Caring for a partner
- ◯ Caring for a parent
- ◯ Caring for another family member (please comment below)
- ◯ Caring for a friend
- ◯ Caring for a neighbour
- ◯ Other (please comment below)

Make a comment on your choice here:

[] Please choose your country of residence.

Choose one of the following answers

Please choose **only one** of the following:

- ◯ Afghanistan
- ◯ Albania
- ◯ Algeria
- ◯ American Samoa
- ◯ Andorra
- ◯ Angola
- ◯ Anguilla
- ◯ Antarctica
- ◯ Antigua and Barbuda

- Argentina
- Armenia
- Aruba
- ** Australia **
- Austria
- Azerbaijan
- Bahamas
- Bahrain
- Bangladesh
- Barbados
- Belarus
- Belgium
- Belize
- Benin
- Bermuda
- Bhutan
- Bolivia
- Bosnia and Herzegovina
- Botswana
- Bouvet Island
- Brazil
- British Indian Ocean Territory
- Brunei Darussalam
- Bulgaria
- Burkina Faso
- Burundi
- Cambodia
- Cameroon
- Canada
- Cape Verde
- Cayman Islands
- Central African Republic
- Chad
- Chile
- ** China **
- Christmas Island
- Cocos Islands
- Colombia
- Comoros
- Congo
- Cook Islands
- Costa Rica
- Cote d'Ivoire
- Croatia
- Cuba
- Cyprus
- Czech Republic
- Denmark
- Djibouti
- Dominica
- Dominican Republic
- Ecuador
- Egypt

- El Salvador
- Equatorial Guinea
- Eritrea
- Estonia
- Ethiopia
- Falkland Islands
- Faroe Islands
- Fiji
- Finland
- France
- French Guiana
- French Polynesia
- Gabon
- Gambia
- Georgia
- Germany
- Ghana
- Gibraltar
- Greece
- Greenland
- Grenada
- Guadeloupe
- Guam
- Guatemala
- Guinea
- Guinea-Bissau
- Guyana
- Haiti
- Heard Island and McDonald Islands
- Honduras
- Hong Kong
- Hungary
- Iceland
- ** India **
- Indonesia
- Iran
- Iraq
- Ireland
- Israel
- Italy
- Jamaica
- Japan
- Jordan
- Kazakhstan
- Kenya
- Kiribati
- Kuwait
- Kyrgyzstan
- Laos
- Latvia
- Lebanon
- Lesotho
- Liberia

- Libya
- Liechtenstein
- Lithuania
- Luxembourg
- Macao
- Madagascar
- Malawi
- Malaysia
- Maldives
- Mali
- Malta
- Marshall Islands
- Martinique
- Mauritania
- Mauritius
- Mayotte
- Mexico
- Micronesia
- Moldova
- Monaco
- Mongolia
- Montenegro
- Montserrat
- Morocco
- Mozambique
- Myanmar
- Namibia
- Nauru
- Nepal
- Netherlands
- Netherlands Antilles
- New Caledonia
- ** New Zealand **
- Nicaragua
- Niger
- Nigeria
- Norfolk Island
- North Korea
- Norway
- Oman
- Pakistan
- Palau
- Palestinian Territory
- Panama
- Papua New Guinea
- Paraguay
- Peru
- Philippines
- Pitcairn
- Poland
- Portugal
- Puerto Rico
- Qatar

- Romania
- Russian Federation
- Rwanda
- Saint Helena
- Saint Kitts and Nevis
- Saint Lucia
- Saint Pierre and Miquelon
- Saint Vincent and the Grenadines
- Samoa
- San Marino
- Sao Tome and Principe
- Saudi Arabia
- Senegal
- Serbia
- Seychelles
- Sierra Leone
- Singapore
- Slovakia
- Slovenia
- Solomon Islands
- Somalia
- South Africa
- South Georgia
- South Korea
- Spain
- Sri Lanka
- Sudan
- Suriname
- Svalbard and Jan Mayen
- Swaziland
- Sweden
- Switzerland
- Syrian Arab Republic
- Taiwan
- Tajikistan
- Tanzania
- Thailand
- The Former Yugoslav Republic of Macedonia
- Timor-Leste
- Togo
- Tokelau
- Tonga
- Trinidad and Tobago
- Tunisia
- Turkey
- Turkmenistan
- Tuvalu
- Uganda
- Ukraine
- United Arab Emirates
- ** United Kingdom **
- ** United States **
- United States Minor Outlying Islands

- Uruguay
- Uzbekistan
- Vanuatu
- Vatican City
- Venezuela
- Vietnam
- Virgin Islands
- Wallis and Futuna
- Western Sahara
- Yemen
- Zambia
- Zimbabwe

[] What is your current income bracket? (In your local currency)

Please choose the appropriate response for each item:

	0 - 20,000	20,001 - 40,000	40,001 - 60,000	60,001 - 80,000	80,001 - 100,000	more than 100,001
Yearly before-tax income	○	○	○	○	○	○

Group 1

[] How many hours per day do you spend managing behavioural and psychological symptoms of dementia (BPSD), i.e. agitation, aggression, depression, physical or verbal behaviours?

Only numbers may be entered in this field.

Please write your answer here:

[ ]

[]
Social assistive technologies in the context of this research are electro-mechanical devices that facilitate social interaction between carers and people with dementia. Social assistive technologies can be in the form of animal-like social robots, telepresence robots (iPad on wheels) and humanoid robots.

Please tick the social assistive technologies that you recognise.

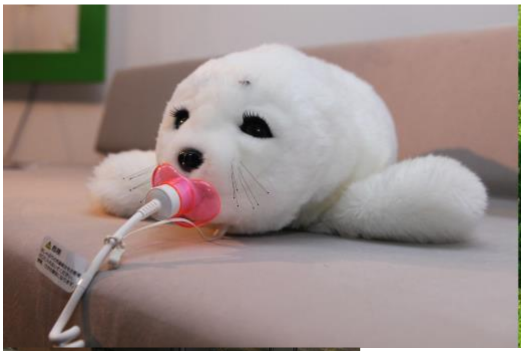
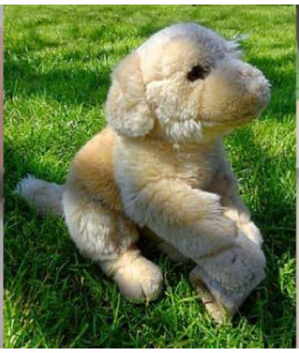
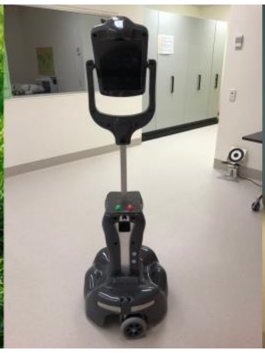
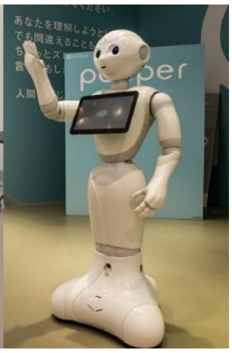
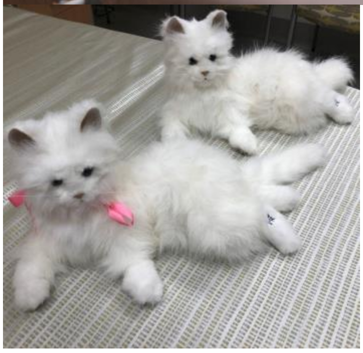

Check all that apply
Please select at most 5 answers

Please choose **all** that apply:

- ☐ PARO-seal
- ☐ Dog-like robots
- ☐ Cat-like robots
- ☐ Giraff
- ☐ Pepper
- ☐ None

[] Have you used any social assistive technologies, social robots or telepresence robots before?

Please choose **only one** of the following:

- ○ Yes
- ○ No

[] In total, how many social assistive technology devices do you currently have available in you?(or if previous carer/RN, previous workplace)? Please choose the name and number of the types being used?

Only answer this question if the following conditions are met:
Answer was 'Yes' at question '9 [FCS08]' (Have you used any social assistive technologies, social robots or telepresence robots before?)

Please choose the appropriate response for each item:

	PARO-seal	Joy for all Dog companion	Joy for all Cat companion	Telepresence robots	Humanoid	Doll-like robot	Other	1	2	3	4	5	6	7	8	9	10 or more
Type and number	○	○	○	○	○	○	○	○	○	○	○	○	○	○	○	○	○
Type and number	○	○	○	○	○	○	○	○	○	○	○	○	○	○	○	○	○
Type and number	○	○	○	○	○	○	○	○	○	○	○	○	○	○	○	○	○
Type and number	○	○	○	○	○	○	○	○	○	○	○	○	○	○	○	○	○
Type and number	○	○	○	○	○	○	○	○	○	○	○	○	○	○	○	○	○
Type and number	○	○	○	○	○	○	○	○	○	○	○	○	○	○	○	○	○
Type and number	○	○	○	○	○	○	○	○	○	○	○	○	○	○	○	○	○

If zero leave the table blank.

[] Please choose the name and owner of the robot?

Only answer this question if the following conditions are met:
Answer was 'Yes' at question '9 [FCS08]' (Have you used any social assistive technologies, social robots or telepresence robots before?)

Please choose the appropriate response for each item:

	PARO-seal	Joy for all Dog companion	Joy for all Cat companion	Telepresence robots	Humanoid	Doll-like robot	Other	Aged care facility	Privately owned by person with dementia	Community care group	Other
Type and owner	○	○	○	○	○	○	○	○	○	○	○
Type and owner	○	○	○	○	○	○	○	○	○	○	○
Type and owner	○	○	○	○	○	○	○	○	○	○	○
Type and owner	○	○	○	○	○	○	○	○	○	○	○
Type and owner	○	○	○	○	○	○	○	○	○	○	○
Type and owner	○	○	○	○	○	○	○	○	○	○	○
Type and owner	○	○	○	○	○	○	○	○	○	○	○

If zero leave the table blank.

[] Have you received any formal training associated with social assistive technologies?

Choose one of the following answers

Please choose **only one** of the following:

- ○ Yes. Please expand below
- ○ No

Make a comment on your choice here:

[]

What level of importance would you rate each of the following barriers to adoption concerning the purchase of social assistive technologies?

Please choose the appropriate response for each item:

	Not Important	Kind of Important	Medium Importance	Somewhat Important	Quite Important	Very Important
Battery life	○	○	○	○	○	○
Hygiene	○	○	○	○	○	○
Training	○	○	○	○	○	○
Cost	○	○	○	○	○	○
Data Security	○	○	○	○	○	○
Maintenance	○	○	○	○	○	○
Perceptions of families	○	○	○	○	○	○
Upgradability	○	○	○	○	○	○
Integration into care activities	○	○	○	○	○	○
Personalisation to the person with dementia	○	○	○	○	○	○
Safety	○	○	○	○	○	○

[]

Please state any ethical considerations for introducing social assistive technologies as a tool for caring for people with dementia? e.g. Dignity and privacy.

Please write your answer here:

[]

"Pepper" is a humanoid robot capable of autonomous navigation, limb movement and speech recognition with verbal dialogue. Pepper also has touch sensors, a display screen for visual information and cameras that can recognise, then interact with the user.

From your experience, how would you rate the following assistive technology in terms of being accepted by people with dementia?

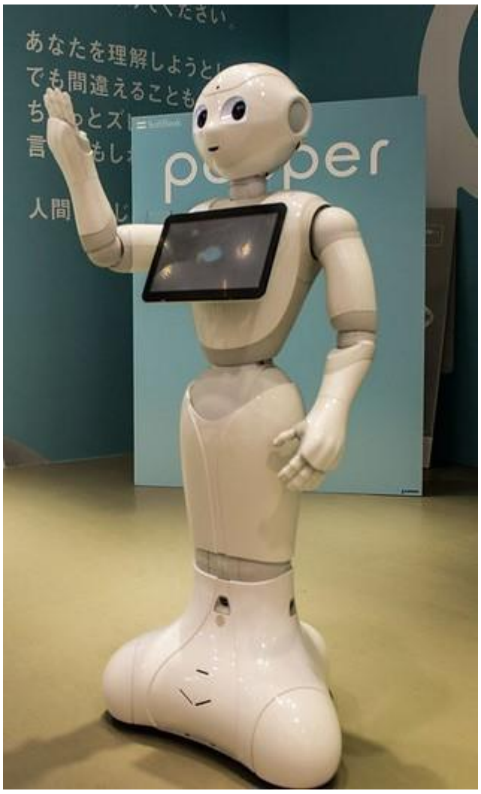

Choose one of the following answers

Please choose **only one** of the following:

- Not likely to be accepted at all
- Maybe accepted by a few
- Likely
- Highly likely
- It may depend on the person
- No opinion

Make a comment on your choice here:

[]

PARO (Personal Assistant Robot), a fur-seal robot, is specifically designed for people living with dementia. PARO has touch, vision, audio, temperature and movement sensors with just as many outputs to match these inputs. PARO is capable of complex emotional interactions.

From your experience, how would you rate the following assistive technology in terms of being accepted by people with dementia?

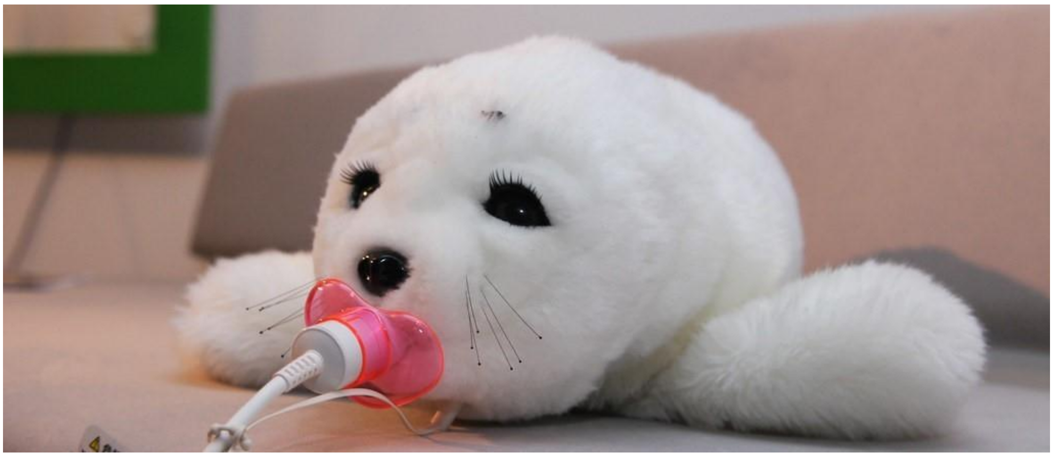

Choose one of the following answers

Please choose **only one** of the following:

- Not likely to be accepted at all
- Maybe accepted by a few
- Likely
- Highly likely
- It may depend on the person
- No opinion

Make a comment on your choice here:

[]

Nao is a humanoid robot that is capable of language interaction and autonomous navigation. Nao specialises in mimicking human movements.

From your experience, how would you rate the following assistive technology in terms of being accepted by people with dementia?

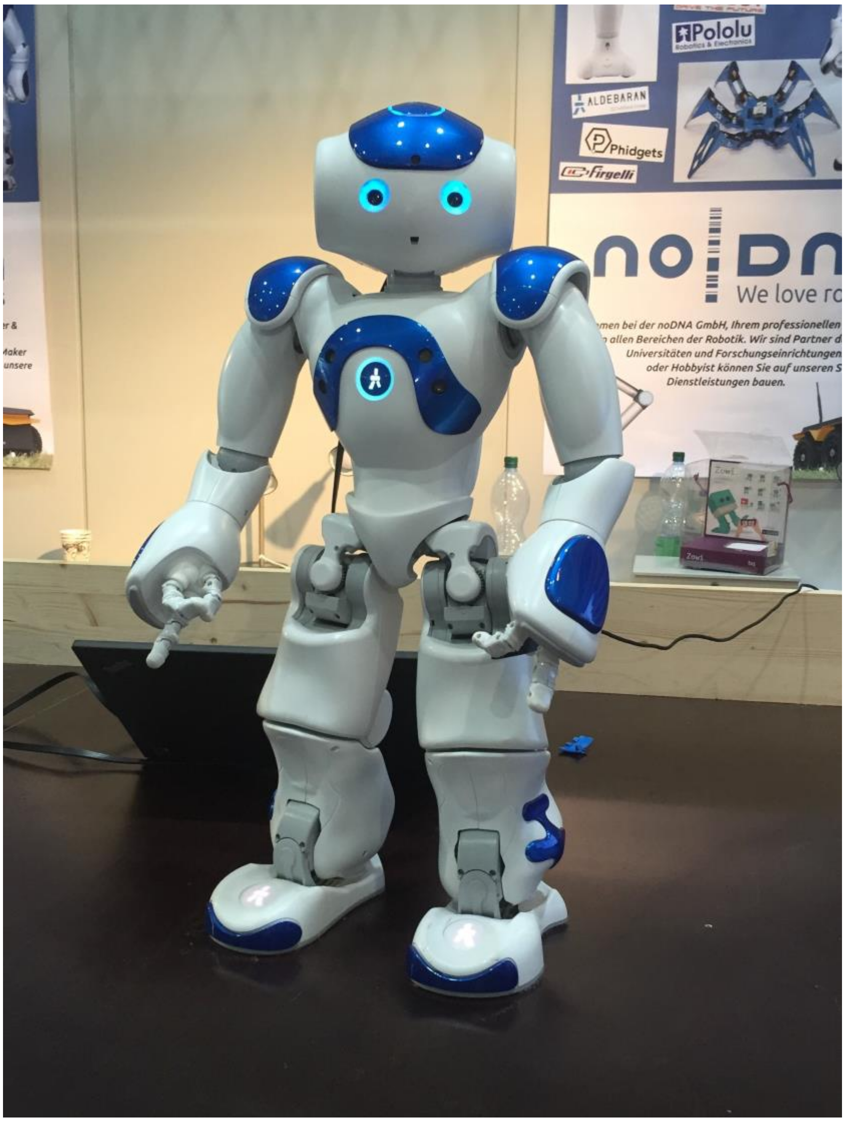

Choose one of the following answers

Please choose **only one** of the following:

- ◯ Not likely to be accepted at all
- ◯ Maybe accepted by a few
- ◯ Likely
- ◯ Highly likely
- ◯ It may depend on the person
- ◯ No opinion

Make a comment on your choice here:

[]

Telepresence robots have a screen and camera that allow people with dementia to interact with health professionals, family, and friends over the internet. Telepresence robots are designed to navigate autonomously if needed.

From your experience, how would you rate the following assistive technology in terms of being accepted by people with dementia?

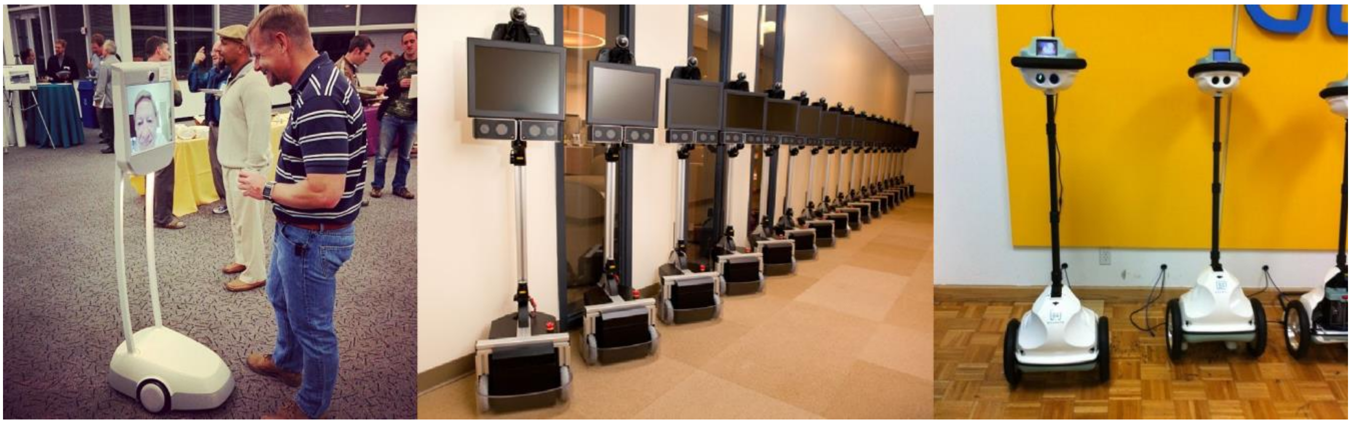

Choose one of the following answers

Please choose **only one** of the following:

- ◯ Not likely to be accepted at all
- ◯ Maybe accepted by a few
- ◯ Likely
- ◯ Highly likely
- ◯ It may depend on the person
- ◯ No opinion

Make a comment on your choice here:

[]

Dog-like robot companions can react to sound and touch by making puppy-like noises and wagging their tails. Some are also capable of head and mouth movements.

From your experience, how would you rate the following assistive technologies in terms of being accepted by people with dementia?

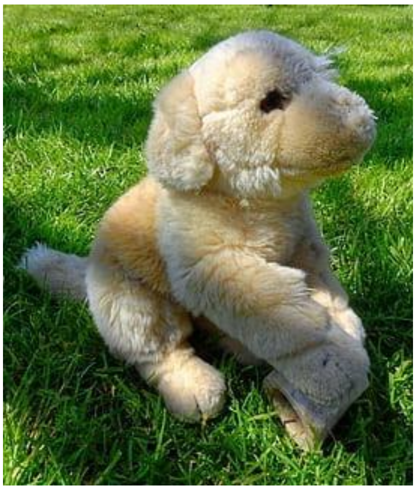

Choose one of the following answers

Please choose **only one** of the following:

- ◯ Not likely to be accepted at all
- ◯ Maybe accepted by a few
- ◯ Likely
- ◯ Highly likely
- ◯ It may depend on the person
- ◯ No opinion

Make a comment on your choice here:

[] Why do **you** consider social assistive technologies to be not useful to extremely useful (0-5)?

Comment only when you choose an answer.
Please select at most one answer

Please choose all that apply and provide a comment:

- ☐ 0 - Not at all Useful
- ☐ 1
- ☐ 2
- ☐ 3
- ☐ 4
- ☐ 5 - Extremely Useful
- ☐ No opinion

Group 2

[] Great work so far!!! You are halfway. We know surveys can be fatiguing. We would like to encourage you to keep going as only full surveys can be used. Thanks again for your time and input.

Choose one of the following answers

Please choose **only one** of the following:

- ◯ Tick the box to continue.

[]
In terms of importance, how would you rate the following attributes?

This question is about designing social assistive technologies that are feasible in the 'real world'.

Please choose the appropriate response for each item:

	Not Important	Kind of Important	Medium Importance	Somewhat Important	Quite Important	Very Important
Acceptability	◯	◯	◯	◯	◯	◯
Demand	◯	◯	◯	◯	◯	◯
Implementation	◯	◯	◯	◯	◯	◯
Practicality	◯	◯	◯	◯	◯	◯
Adaptation	◯	◯	◯	◯	◯	◯
Integration	◯	◯	◯	◯	◯	◯
Expansion	◯	◯	◯	◯	◯	◯
Effectiveness testing	◯	◯	◯	◯	◯	◯

[]How long do you perceive typical interactions will last between people with dementia and social assistive technologies?

Please choose the appropriate response for each item:

	0-5min	6-10min	11-15min	16-20min	21-25min	25min and over
Early-stage dementia	◯	◯	◯	◯	◯	◯
Mid-stage dementia	◯	◯	◯	◯	◯	◯
Severe dementia	◯	◯	◯	◯	◯	◯

[]
How would you rate the following training modes as a means to learn the operational procedures of assistive technologies?

Please choose the appropriate response for each item:

	1 Least Preferred	2	3	4	5 Most Preferred
In-person training	◯	◯	◯	◯	◯
Training online (interactive)	◯	◯	◯	◯	◯
Training using videos only from a manufacturer	◯	◯	◯	◯	◯

[]
If designing a social assistive device for a person with mid-to-late-stage dementia, what importance would you place on the following Input features?

Please choose the appropriate response for each item:

	Not Important	Kind of Important	Medium Importance	Quite Important	Very Important
**Vision**: Face position tracking (no face recognition), so assistive technologies can point in the direction of the user	◯	◯	◯	◯	◯
**Vision:** Face recognition to identify moods such as happy or sad	◯	◯	◯	◯	◯
**Vision:** Face recognition to identify pain	◯	◯	◯	◯	◯
**Vision:** Face recognition as a way to remember each person for future interaction	◯	◯	◯	◯	◯
**Vision:** Light sensors for complex sleep tracking purposes	◯	◯	◯	◯	◯
**Audio:** Pitch and Tone only to analyse emotions such as happiness or anger	◯	◯	◯	◯	◯
**Audio:** Speech recognition for verbal conversations between a person with dementia, and an assistive technology	◯	◯	◯	◯	◯
**Audio:** Song recognition for personalised interactions (so a device can sing along to a song using synthetic voices) or play the song	◯	◯	◯	◯	◯
**Audio:** Song recognition for personalised interactions (so a device can download then play a certain song)	◯	◯	◯	◯	◯
**Touch:** Stroking or animal patting sensors	◯	◯	◯	◯	◯
**Touch:** Temperature sensors (information to be relayed to the carer)	◯	◯	◯	◯	◯
**Touch:** Temperature sensors to be used by internal programming to sense discomfort	◯	◯	◯	◯	◯
**Touch:** Heartrate monitor (information to be relayed to the carer)	◯	◯	◯	◯	◯

	Not Important	Kind of Important	Medium Importance	Quite Important	Very Important
**Touch:** Heartrate monitor to be used by internal programming to sense agitation or pain	○	○	○	○	○
**Movement:** : Acceleration for measuring aggression	○	○	○	○	○
**Movement:** Location monitoring	○	○	○	○	○

[]

Are there any other input features that should be considered for social assistive technologies, or any items from the previous question that you would like to provide further comment on?

Please write your answer here:

[]

If designing a social assistive device for a person with mid-to-late-stage dementia, what importance would you place on the following Output features? (these features may be turned off or on depending on the person)

Please choose the appropriate response for each item:

	Not Important	Kind of Important	Medium Importance	Quite Important	Very Important
**Vision:** Moving eyebrows	○	○	○	○	○
**Vision:** Smile or frowning mouth	○	○	○	○	○
**Vision:** Ears that move	○	○	○	○	○
**Vision:** Blinking eyes	○	○	○	○	○
**Audio:** If a social assistive device is made to resemble an animal e.g. if a device resembles a dog, it should bark	○	○	○	○	○
**Audio:** If a social assistive device is made to resemble a human or mechanised robot, it should use verbal English to communicate	○	○	○	○	○
**Audio:** Music should be considered as an output on social assistive technologies that resemble animals (cats, dogs)	○	○	○	○	○
**Audio:** Music should be considered as an output on social assistive technologies that resemble living entities	○	○	○	○	○
**Audio:** Music should be considered as an output on social assistive technologies that resemble mechanised robots	○	○	○	○	○
**Audio:** There should be an audible heartbeat	○	○	○	○	○
**Touch:** It would be advantageous to heat the surface of an assistive technology above 30°Celcius	○	○	○	○	○
**Touch:** Vibration features should be available as a feature in all social assistive technologies (not just purring cats)	○	○	○	○	○
**Touch:** Vibrating heartbeats should be a feature in social assistive technologies that resemble living entities	○	○	○	○	○

[] Are there any other Output features that should be considered for social assistive devices, or any items from the previous question that you would like to provide further comment on?

Please write your answer here:

Group 3

[]

To what extent do you agree or disagree with the following statements?

Please choose the appropriate response for each item:

	0 - Strongly Disagree	2	3	4	5 - Strongly Agree
Social assistive technologies should be made to look like animals	○	○	○	○	○
Social assistive technologies should be made to look like Humans	○	○	○	○	○
Social assistive technologies should be made to look like mechanised robots	○	○	○	○	○
Social assistive technologies should have coloured LED lights for sensory stimulation	○	○	○	○	○
Social assistive technologies should be able to walk or drive independent of human operators	○	○	○	○	○
Social assistive technologies should have simple head movement only	○	○	○	○	○
Social assistive technologies should be able to move limbs and tails, including the head	○	○	○	○	○
Social assistive technologies need only to be passive in their movement	○	○	○	○	○

[]

Research has shown that data privacy is an important topic when designing social assistive technologies. Please provide your opinion on each of the following levels of data collection.

Please choose the appropriate response for each item:

	Not acceptable	Borderline Acceptable	Acceptable	No opinion
Instant sensory responses - no data recorded	○	○	○	○
Data recorded of how many times each response is used (e.g. a person patting a robotic cat and the cat purring is one action). This can be displayed after an interaction but will need to be manually recorded and reset by the carer.	○	○	○	○
Recorded data recognises different persons by assigning pseudonyms (i.e. person one, person three). Identification happens via voice or facial recognition and is used to turn features on or off (e.g. vocal output) for that person. No hardware option would be available to access data and would be automatically deleted on a periodic basis.	○	○	○	○
Recorded data recognises different persons by assigning pseudonyms (i.e. person one, person three) and records enough data to build a database on a person's normal facial and audio information. This information can be used to identify pain, discomfort, or negative emotions. This data would need to have a central repository that is secure and be operated offline.	○	○	○	○
Data is recorded on a person's favourite music, movies, colours, voice, facial and location information. The data will be used for personalised interactions with an individual. Pseudonyms would still be used, and data would be kept on a secure central repository. For this level of sophisticated interaction, an internet connection is required to retrieve information, like an audio file of a person's favourite song.	○	○	○	○

[]

Research has shown that data privacy is an important topic when designing social assistive technologies. Please provide your opinion on each of the following levels of data collection.

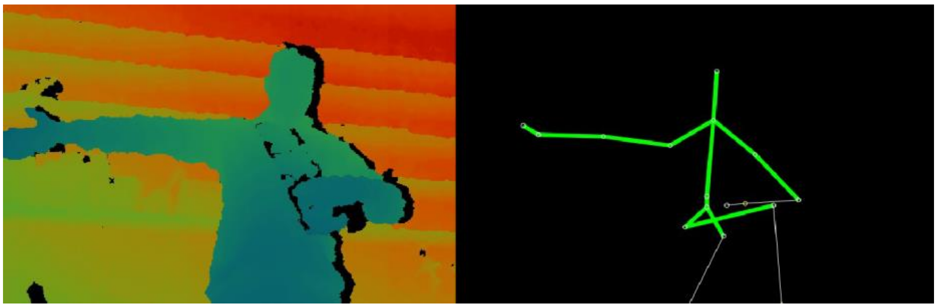

Please choose the appropriate response for each item:

	Not acceptable	Borderline Acceptable	Acceptable	No opinion
Monitoring a person with dementia is achieved by an infrared camera that only shows outlines and colour. This would only be used when there is a high risk of falls or other injurys.	○	○	○	○
Monitoring a person with dementia is achieved by a tracking camera that shows body motion (without identifiable visual video). This would only be used when there is a high risk of falls, other injury or wandering.	○	○	○	○
Monitoring a person with dementia is achieved by an infrared camera that only shows outlines and colour. This would be used in personal rooms of residential aged care facilities where people with dementia reside regardless of the risk of self-injury.	○	○	○	○

	Not acceptable	Borderline Acceptable	Acceptable	No opinion
Monitoring a person with dementia is achieved by a tracking camera that shows body motion (without identifiable visual video). This would be used in personal rooms of residential aged care facilities where people with dementia reside regardless of the risk of the self-injury.	○	○	○	○

[]
Is there any important area that you think this survey has not mentioned?

Please write your answer here:

[] What would you like to see added to a social assistive device?

Please write your answer here:

[]
Did you hear about this survey from Stepup for Dementia? Y or N

Please write your answer here:

[] Is there anything you would like to add that can help us in our research?

Please write your answer here:
