# Supplementary material for: Social robot design preferences as reported by stakeholders
Source: Front Dement. 2026 May 18;5:1821891. doi: 10.3389/frdem.2026.1821891 (PMC13222834; doi:10.3389/frdem.2026.1821891)
Supplement: Supplementary file 4 [file Supplementary_file_4.pdf]

Care providers input for the design of social assistive technology for people with dementia

Care providers input for “Bespoke social-assistive technologies for people with dementia: A user-driven approach."

Basic Demographics

[]

Are you an NDIS provider, RACP or person responsible for purchasing technology in the care provider group?

Choose one of the following answers

Please choose **only one** of the following:

- ◯ NDIS
- ◯ RACF
- ◯ Person responsible for purchasing technology in the care provider group
- ◯ Other (please expand below)

Make a comment on your choice here:

[]
What is your position within this organisation?

Choose one of the following answers

Please choose **only one** of the following:

- RACF manager
- Duty manager
- NDIS provider
- Regional manager
- Company Manager
- Other (please comment below)

Make a comment on your choice here:

[]
Gender

Choose one of the following answers

Please choose **only one** of the following:

- Female
- Male
- Transgender
- Gender not specified

[]
Which of the following groups does your organisation best fit?

Choose one of the following answers

Please choose **only one** of the following:

- Private System
- Public System

[] Please choose your country of residence.

Choose one of the following answers

Please choose **only one** of the following:

- Afghanistan
- Albania

- Algeria
- American Samoa
- Andorra
- Angola
- Anguilla
- Antarctica
- Antigua and Barbuda
- Argentina
- Armenia
- Aruba
- ** Australia **
- Austria
- Azerbaijan
- Bahamas
- Bahrain
- Bangladesh
- Barbados
- Belarus
- Belgium
- Belize
- Benin
- Bermuda
- Bhutan
- Bolivia
- Bosnia and Herzegovina
- Botswana
- Bouvet Island
- Brazil
- British Indian Ocean Territory
- Brunei Darussalam
- Bulgaria
- Burkina Faso
- Burundi
- Cambodia
- Cameroon
- Canada
- Cape Verde
- Cayman Islands
- Central African Republic
- Chad
- Chile
- ** China **
- Christmas Island
- Cocos Islands
- Colombia
- Comoros
- Congo
- Cook Islands
- Costa Rica
- Cote d'Ivoire
- Croatia
- Cuba
- Cyprus

- ◯ Czech Republic
- ◯ Denmark
- ◯ Djibouti
- ◯ Dominica
- ◯ Dominican Republic
- ◯ Ecuador
- ◯ Egypt
- ◯ El Salvador
- ◯ Equatorial Guinea
- ◯ Eritrea
- ◯ Estonia
- ◯ Ethiopia
- ◯ Falkland Islands
- ◯ Faroe Islands
- ◯ Fiji
- ◯ Finland
- ◯ France
- ◯ French Guiana
- ◯ French Polynesia
- ◯ Gabon
- ◯ Gambia
- ◯ Georgia
- ◯ Germany
- ◯ Ghana
- ◯ Gibraltar
- ◯ Greece
- ◯ Greenland
- ◯ Grenada
- ◯ Guadeloupe
- ◯ Guam
- ◯ Guatemala
- ◯ Guinea
- ◯ Guinea-Bissau
- ◯ Guyana
- ◯ Haiti
- ◯ Heard Island and McDonald Islands
- ◯ Honduras
- ◯ Hong Kong
- ◯ Hungary
- ◯ Iceland
- ◯ ** India **
- ◯ Indonesia
- ◯ Iran
- ◯ Iraq
- ◯ Ireland
- ◯ Israel
- ◯ Italy
- ◯ Jamaica
- ◯ Japan
- ◯ Jordan
- ◯ Kazakhstan
- ◯ Kenya
- ◯ Kiribati

- Kuwait
- Kyrgyzstan
- Laos
- Latvia
- Lebanon
- Lesotho
- Liberia
- Libya
- Liechtenstein
- Lithuania
- Luxembourg
- Macao
- Madagascar
- Malawi
- Malaysia
- Maldives
- Mali
- Malta
- Marshall Islands
- Martinique
- Mauritania
- Mauritius
- Mayotte
- Mexico
- Micronesia
- Moldova
- Monaco
- Mongolia
- Montenegro
- Montserrat
- Morocco
- Mozambique
- Myanmar
- Namibia
- Nauru
- Nepal
- Netherlands
- Netherlands Antilles
- New Caledonia
- ** New Zealand **
- Nicaragua
- Niger
- Nigeria
- Norfolk Island
- North Korea
- Norway
- Oman
- Pakistan
- Palau
- Palestinian Territory
- Panama
- Papua New Guinea
- Paraguay

- Peru
- Philippines
- Pitcairn
- Poland
- Portugal
- Puerto Rico
- Qatar
- Romania
- Russian Federation
- Rwanda
- Saint Helena
- Saint Kitts and Nevis
- Saint Lucia
- Saint Pierre and Miquelon
- Saint Vincent and the Grenadines
- Samoa
- San Marino
- Sao Tome and Principe
- Saudi Arabia
- Senegal
- Serbia
- Seychelles
- Sierra Leone
- Singapore
- Slovakia
- Slovenia
- Solomon Islands
- Somalia
- South Africa
- South Georgia
- South Korea
- Spain
- Sri Lanka
- Sudan
- Suriname
- Svalbard and Jan Mayen
- Swaziland
- Sweden
- Switzerland
- Syrian Arab Republic
- Taiwan
- Tajikistan
- Tanzania
- Thailand
- The Former Yugoslav Republic of Macedonia
- Timor-Leste
- Togo
- Tokelau
- Tonga
- Trinidad and Tobago
- Tunisia
- Turkey
- Turkmenistan

- Tuvalu
- Uganda
- Ukraine
- United Arab Emirates
- ** United Kingdom **
- ** United States **
- United States Minor Outlying Islands
- Uruguay
- Uzbekistan
- Vanuatu
- Vatican City
- Venezuela
- Vietnam
- Virgin Islands
- Wallis and Futuna
- Western Sahara
- Yemen
- Zambia
- Zimbabwe

[]

What is the highest level of education you have achieved?

Choose one of the following answers

Please choose **only one** of the following:

- Up to and including Year 10
- Year 11 or 12
- Advanced Diploma, Diploma or Cert III/IV
- Bachelor’s degree
- Postgraduate degree or higher

Group 1

[] How many hours per day would an employee typically spend managing behavioural and psychological symptoms of dementia (BPSD), i.e. agitation, aggression, depression, physical or verbal behaviours?

Only numbers may be entered in this field.

Please write your answer here:

[ ]

[]

Social assistive technologies in the context of this research are electro-mechanical devices that facilitate social interaction between carers and people with dementia. Social assistive technologies can be in the form of animal-like social robots, telepresence robots (iPad on wheels) and humanoid robots.

Please tick the social assistive technologies that you recognise.

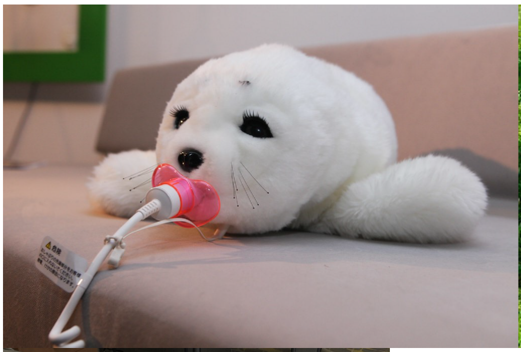
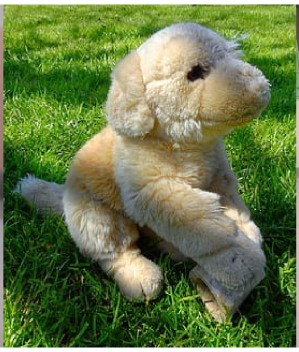
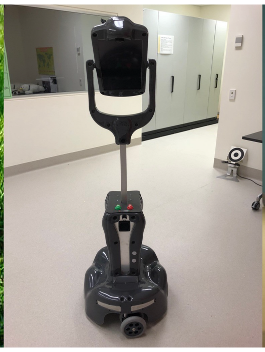
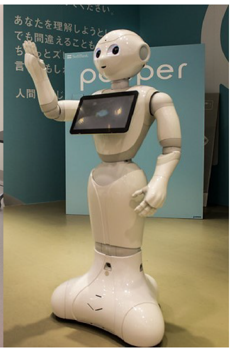
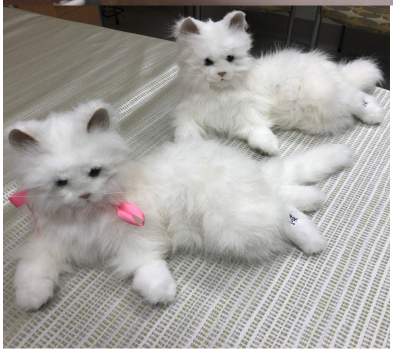

Check all that apply
Please select at most 5 answers

Please choose **all** that apply:

- ☐ PARO-seal
- ☐ Dog-like robots
- ☐ Cat-like robots
- ☐ Giraff
- ☐ Pepper
- ☐ None

[]

Have the carers you employ used any social assistive technologies such as social robots or telepresence robots previously?

Please choose **only one** of the following:

- ◯ Yes
- ◯ No

[]In total, how many social assistive technology devices do you have available in your workplace (or if previous carer/RN, previous workplace)? Please choose the name and number of the types being used?

Only answer this question if the following conditions are met:
Answer was 'Yes' at question '9 [CP08]' (Have the carers you employ used any social assistive technologies such as social robots or telepresence robots previously?)

Please choose the appropriate response for each item:

	PARO-seal	Joy for all Dog companion	Joy for all Cat companion	Telepresence robots	Humanoid	Doll-like robot	Other	1	2	3	4	5	6	7	8	9	10 or more
Type and number																	
Type and number																	
Type and number																	
Type and number																	
Type and number																	
Type and number																	
Type and number																	

If zero leave the table blank.

[] Please choose the name and owner of the robot?

Only answer this question if the following conditions are met:
Answer was 'Yes' at question '9 [CP08]' (Have the carers you employ used any social assistive technologies such as social robots or telepresence robots previously?)

Please choose the appropriate response for each item:

	PARO-seal	Joy for all Dog companion	Joy for all Cat companion	Telepresence robots	Humanoid	Doll-like robot	Other	Aged care facility	Privately owned by person with dementia	Community care group	Other
Type and owner											
Type and owner											
Type and owner											
Type and owner											
Type and owner											
Type and owner											
Type and owner											

If zero leave the table blank.

[]Why do you consider social assistive technologies to be not useful to extremely useful (0-5)?

Comment only when you choose an answer.
Please select at most one answer

Please choose all that apply and provide a comment:

☐ 0 - Not at all Useful

☐ 1

☐ 2

☐ 3

☐ 4

☐ 5 - Extremely Useful

☐ No opinion

[]
What underlying computer operating systems does your facility use? (If appropriate, what is the name of your customised software?)

Comment only when you choose an answer.

Please choose all that apply and provide a comment:

☐ Windows

☐ Ios (Apple)

☐ Linux

☐ Custom

☐ Custom program that runs on Windows

☐ Custom program that runs on Ios (Apple)

☐ Custom program that runs on Linux

[]Does your facility have internet coverage?

Please choose the appropriate response for each item:

	Very poor	Poor	Average	Good	Very Good
Wifi	○	○	○	○	○
Networked (wired)	○	○	○	○	○
Cell coverage	○	○	○	○	○
Internet Security	○	○	○	○	○

Group 2

[] Great work so far!!! You are halfway. We know surveys can be fatiguing. We would like to encourage you to keep going as only full surveys can be used. Thanks again for your time and input.

Choose one of the following answers

Please choose **only one** of the following:

- Tick the box to continue.

[] In your opinion, do people with dementia have a preference for shared or personal devices?

	Please enter your comment here as to why people with dementia have a preference for shared or personal devices.
Shared	
Personal	

This question asks about the use of social assistive technology like social robots, telepresence robots (iPad on wheels), humanoid robots or any other social assistive technology involved in the care of people with dementia.

[]
In your opinion, do carers (professional or informal) have a preference for shared or personal devices?

	Please enter your comment here as to why people with dementia have a preference for shared or personal devices.
Shared	
Personal	

This question asks about the use of social assistive technology like social robots, telepresence robots (iPad on wheels), humanoid robots or any other social assistive technology involved in the care of people with dementia.

[] Do you have a preference for shared or personal devices?

	Please enter your comment as to why you prefer shared or personal devices.
Shared	
Personal	

This question asks about the use of social assistive technology like social robots, telepresence robots (iPad on wheels), humanoid robots or any other social assistive technology involved in the care of people with dementia.

[]
In terms of importance, how would you rate the following attributes?

This question is about designing social assistive technologies that are feasible in the 'real world'.

Please choose the appropriate response for each item:

	Not Important	Kind of Important	Medium Importance	Somewhat Important	Quite Important	Very Important
Acceptability	○	○	○	○	○	○
Demand	○	○	○	○	○	○
Implementation	○	○	○	○	○	○
Practicality	○	○	○	○	○	○
Adaptation	○	○	○	○	○	○
Integration	○	○	○	○	○	○
Expansion	○	○	○	○	○	○
Effectiveness testing	○	○	○	○	○	○

[]
Do you have a procedure for infection control of robots?

If yes, what is the procedure and is it validated?

Choose one of the following answers

Please choose **only one** of the following:

- Yes. Please expand below
- No
- Not exactly. Please expand below

Make a comment on your choice here:

[]

When purchasing equipment like social assistive technologies, who is the authoriser for the following amounts?

Example:

	Approver
$0-500	Facility Manager
$501-1000	Facility Manager
$1001-1500	National head office

	Approver
$0-500	
$501-1000	
$1001-1500	
$1501-3000	
$3001-6000	
$6000 and above	

Group 3

[]
How important would you rate the following barriers to adoption of social assistive technologies?

Please choose the appropriate response for each item:

	Not Important	Kind of Important	Medium Importance	Somewhat Important	Quite Important	Very Important
Battery life	○	○	○	○	○	○
Hygiene	○	○	○	○	○	○
Training	○	○	○	○	○	○
Cost	○	○	○	○	○	○
Data Security	○	○	○	○	○	○
Maintenance	○	○	○	○	○	○
Perceptions of families	○	○	○	○	○	○
Upgradability	○	○	○	○	○	○
Integration into care activities	○	○	○	○	○	○
Personalisation to the person with dementia	○	○	○	○	○	○
Safety	○	○	○	○	○	○

[]
Please state any ethical considerations for introducing social assistive technologies as a tool for caring for people with dementia? e.g. Dignity and privacy.

Please write your answer here:

[]
How would you rate the following training as a means to learn the operational procedures of social assistive technologies (no prerequisites required, and carers will be paid for their time)?

Please choose the appropriate response for each item:

	1 Least appealing	2	3	4	5 Most appealing
In-house training at work	○	○	○	○	○
Training online (interactive)	○	○	○	○	○
Training using videos only from a manufacturer	○	○	○	○	○

[]Research has shown that data privacy is an important topic when designing social assistive technologies. Please provide your opinion on each of the following levels of data collection.

Please choose the appropriate response for each item:

	Not acceptable	Borderline Acceptable	Acceptable
Instant sensory responses - no data recorded	○	○	○
Data recorded of how many times each response is used (e.g. a person patting a robotic cat and the cat purring is a single action). The data can be displayed after an interaction but will need to be manually recorded and reset by the carer.	○	○	○
Recorded data recognises different persons by assigning pseudonyms (i.e. person one, person three). Identification happens via voice or facial recognition and is used to turn features on or off (e.g. vocal output) for that person. No hardware option would be available to access data and would be automatically deleted on a periodic basis.	○	○	○
Recorded data recognises different persons by assigning pseudonyms (i.e. person one, person three) and records enough data to build a database on a person’s normal facial and audio information. This information can be used to identify pain, discomfort, or negative emotions. This data would need to have a secure central repository and be operated offline.	○	○	○
Data is recorded on a person’s favourite music, movies, colours, voice, facial and location information. The data will be used for personalised interactions with an individual. Pseudonyms would still be used, and data would be kept on a secure central repository. For this level of sophisticated interaction, an internet connection is required to retrieve information, like an audio file of a person's favourite song.	○	○	○

[]
Research has shown that data privacy is an important topic when designing social assistive technologies. Please provide your opinion on each of the following levels of data collection.

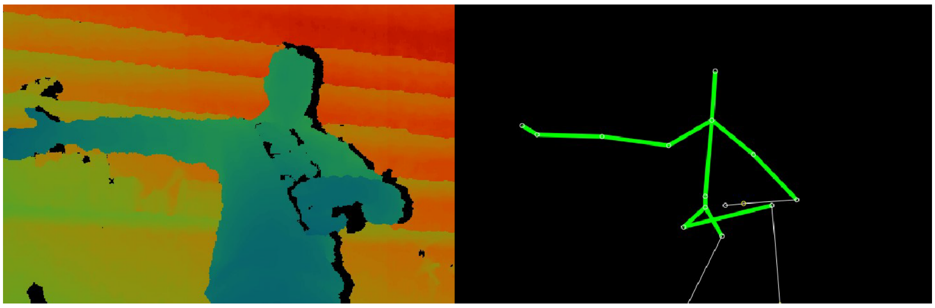

Please choose the appropriate response for each item:

	Not acceptable	Borderline Acceptable	Acceptable
Monitoring a person with dementia is achieved by an infrared camera that shows outlines and colour. This would only be used when there is a high risk of a fall or other injuries.	○	○	○
Monitoring a person with dementia is achieved by a tracking camera that shows body motion (without identifiable visual video). This would only be used when there is a high risk of injuries or wandering.	○	○	○
Monitoring a person with dementia is achieved by an infrared camera that only shows outlines and colour. This would be used in personal rooms of residential aged care facilities where people with dementia reside regardless of the risk of injury.	○	○	○
Monitoring a person with dementia is achieved by a Skeletal tracking camera that only shows limb position. This would be used in personal rooms of residential aged care facilities where people with dementia reside regardless of the risk of injury.	○	○	○

[]
Is there any important area that you think this survey has not mentioned?

Please write your answer here:

[]Is there anything you would like to add that can help us in our research?

Please write your answer here:
